# Pathology in Captive Wild Felids at German Zoological Gardens

**DOI:** 10.1371/journal.pone.0130573

**Published:** 2015-06-18

**Authors:** Johannes Junginger, Florian Hansmann, Vanessa Herder, Annika Lehmbecker, Martin Peters, Martin Beyerbach, Peter Wohlsein, Wolfgang Baumgärtner

**Affiliations:** 1 Department of Pathology, University of Veterinary Medicine, Hannover, Lower Saxony, Germany; 2 Center for Systems Neuroscience, Hannover, Lower Saxony, Germany; 3 Chemisches und Veterinäruntersuchungsamt Westphalia, Arnsberg, North Rhine-Westphalia, Germany; 4 Department of Biometry, Epidemiology and Information Processing, University of Veterinary Medicine, Hannover, Lower Saxony, Germany; Colorado State University, UNITED STATES

## Abstract

This retrospective study provides an overview on spontaneous diseases occurring in 38 captive wild felids submitted for necropsy by German zoological gardens between 2004 and 2013. Species included 18 tigers, 8 leopards, 7 lions, 3 cheetahs and 2 cougars with an age ranging from 0.5 to 22 years. Renal lesions, predominantly tubular alterations (intra-tubular concrements, tubular degeneration, necrosis, intra-tubular cellular debris, proteinaceous casts, dilated tubuli) followed by interstitial (lympho-plasmacytic inflammation, fibrosis, metastatic-suppurative inflammation, eosinophilic inflammation) and glomerular lesions (glomerulonephritis, glomerulosclerosis, amyloidosis) were detected in 33 out of 38 animals (87%). Tumors were found in 19 of 38 felids (50%) with 12 animals showing more than one neoplasm. The tumor prevalence increased with age. Neoplasms originated from endocrine (11), genital (8), lympho-hematopoietic (5) and alimentary organs (4) as well as the mesothelium (3). Most common neoplasms comprised uterine/ovarian leiomyomas (5/2), thyroid adenomas/adenocarcinoma (5/1), pleural mesotheliomas (3), hemangiosarcomas (2) and glossal papillomas (2). Inflammatory changes were frequently encountered in the intestine and the lung. Two young animals displayed metastatic mineralization suggestive of a vitamin D- or calcium intoxication. One tiger exhibited degenerative white matter changes consistent with an entity termed large felid leukoencephalomyelopathy. Various hyperplastic, degenerative and inflammatory changes with minor clinical significance were found in several organs. Summarized, renal lesions followed by neoplastic changes as well as inflammatory changes in lung and gastrointestinal tract represent the most frequent findings in captive wild felids living in German zoological gardens.

## Introduction

Wild felid populations are rapidly decreasing in their natural habitat due to various factors including comprehensive biosphere changes, poaching, and exposure to infectious agents [1,2]. With regard to these emerging changes in wild felid biodiversity, many of them are classified as endangered species by the International Union for Conservation of Nature and Natural Resources, IUCN [3]. In parallel, many wild felids are housed in zoological gardens worldwide and represent a theoretical source of genetic material to recruit animals for reintroduction into the wild [4]. These collections may have high stability due to individual nursing and life-long monitoring [5,6]. However, they can be affected by several disorders triggered by environmental factors, genetic changes and infectious agents [1,5,7]. Therefore, knowledge about the frequency of diseases, including those in aged individuals, is important for clinicians, biologists and pathologists.

Neoplastic diseases may cause high morbidity and mortality in several captive wildlife species. An increased rate of neoplasms may further be indicative of the presence of infectious agents, genetic aberrations or an adverse environment [8,9]. In captive wild felids, a wide range of different neoplasms has been described, most of which are reported as single cases [10–14]. Only a few studies focused on tumor prevalence in larger populations of wild felids and all publically available data originate from zoological gardens in the United States [5,6,15]. Chronic nephropathy is common in aged domestic and non-domestic cats [16] and might also constitute an issue in geriatrics of captive wild felids.

In addition to neoplastic, inflammatory and degenerative lesions, infectious agents, including feline leukemia virus (FeLV), feline immunodeficiency virus (FIV), feline infectious peritonitis virus (FIPV) and *Helicobacter* sp., represent important pathogens in captive and wild felid populations [8,17–19]. FIV has been described in lions, cheetahs, panthers, cougars, jaguars, leopards and in a tiger [17,20–22]. It is suggested, that FIV adaptation in many exotic felids started within the last 10,000 to 20,000 years [21]. Investigation of FIV infection in free ranging lions in Botswana and Tanzania revealed an immunosuppression resulting in lymphadenopathy, gingivitis, tongue papillomas, dehydration and poor coat condition [23]. Similar changes are described in FIV positive domestic cats [24]. Furthermore, FIV infection was associated with malignant lymphoma in one African lion [25]. Simultaneously, this animal suffered from pneumonia, neutropenia and thrombocytopenia [25]. Interestingly, no FIV infection was observed in 11 lions suffering from malignant lymphoma in another study [26].

In contrast to FIV, infection with FeLV seems to be more restricted to domestic cats as it rarely occurs in non-domestic felids [21]. FeLV infection detected by polymerase chain reaction was associated with a multicentric T cell lymphoma in one cheetah [8]. In addition, FeLV antigen was detected in 1 of 11 African lions suffering from malignant lymphoma as determined by immunohistochemistry [26]. Therefore, Harrison et al. [26] assumed a minor pathogenetic relevance of FeLV in lions. This is in contrast to its domestic counterpart. Interestingly, FeLV neutralizing antibodies but no FeLV antigen were found in a 12 years old male clouded leopard [27]. FeLV antibodies are also demonstrated in Florida panthers and an association with clinical signs including lymphadenopathy, anemia, septicemia and weight loss is suggested [28,29].

The aims of the present study are a) to characterize frequently observed gross and histopathological findings including neoplastic, degenerative and inflammatory lesions in wild felids kept in German zoological gardens, b) to highlight rare salient lesions and c) to give a special focus on age-related changes.

## Materials and Methods

### Study design

Data of 38 captive wild felids originating from eight German zoological gardens were reviewed retrospectively. Animals included 18 tigers (*Panthera tigris*), 8 leopards (*Panthera pardus*), 7 lions (*Panthera leo*), 3 cheetahs (*Acinonyx jubatus*) and 2 cougars (*Puma concolor*) comprising 24 female and 14 male individuals with age ranging from 0.5 to 22 years (median: 13 years). Except cougars, animal species were housed in more than one zoological garden. The animals were necropsied between 2004 and 2013 at the Department of Pathology, University of Veterinary Medicine Hannover, and Staatliches Veterinäruntersuchungsamt Arnsberg, North Rhine-Westphalia. The time between death and post-mortem examination ranged from 0 to 5 days with a median time interval of 1 day (S1 Table). The body condition was scored as obese, good, moderate, poor and cachectic. Animals in a good body condition revealed physiological amounts of subcutaneous and abdominal fat tissue, whereas animals with a moderate body condition showed reduced amounts of subcutaneous but only very low abdominal fat tissue. Animals in a poor body condition possessed only limited amounts of fat reserves frequently associated with generalized muscle atrophy. In contrast cachectic animals lacked fat reserves and displayed a serous atrophy of the bone marrow or coronal myocardial fat tissue. Detailed information on each animal concerning species, gender and age is given in Table 1 and S1 Table. This study was carried out in accordance to the German animal welfare act. All animals were dead at the time of submission for necropsy and the authors confirm that no animals were sacrificed for the purpose of this study. Post-mortem examinations were carried out by order of the operators of the zoological gardens to investigate the causes of illness or death. This retrospective study was not approved by an ethics committee since it is not an animal experiment and all necropsies were performed as recommended or requested by the following legal provisions applicable for zoological gardens in the Federal Republic of Germany, respectively: “Gutachten über die Mindestanforderungen zur Haltung von Säugetieren” (German Federal Ministry of Food and Agriculture, 2014), Council Directive 92/65/EEC, Council Directive 1999/22/EC.

**Table 1:** Animals used in this study.

Animal number	Species	Sex	Age (years)	Nutritional status	FIV / FeLV	Urea (mg/dl)	Significant pathological findings	Manner of death
1	Cheetah (Acinonyx jubatus)	M	10	Poor	- / -	120	Pleura: mesothelioma; spleen: myelolipoma	Spontaneous
2	Cheetah (Acinonyx jubatus)	F	2	Good	- / -	n.d.	Hemorrhage (presumably due to trauma); cardiac lipomatosis	Spontaneous
3	Cheetah (Acinonyx jubatus)	F	11	Good	- / -	*	GN, IN, pyelitis, TD, renal intratubular concrements; hemorrhagic gastroenteritis; melena; cardiac lipomatosis	Euthanasia
4	Cougar (Puma concolor)	F	18	Good	- / -	150	GN, IN; thyroid gland: adenocarcinoma; kidney, liver, right femoral bone, bone marrow: hemangiosarcoma; cerebral meninges: psammomatous meningioma; skin: T cell lymphoma; parathyroid hyperplasia; cardiac lipomatosis; chronic lymphohistiocytic pododermatitis	Euthanasia
5	Cougar (Puma concolor)	M	14	Good	- / -	260	GN, IN, pyelitis, renal intratubular concrements; suppurative pneumonia; parathyroid gland: bilateral hyperplasia; exocrine pancreas: nodular hyperplasia	Euthanasia
6	Leopard (Panthera pardus)	M (n)	18	Poor	- / -	150	GN, IN; pituitary gland: carcinoma; thyroid gland: cystic adenoma; pancreatic cysts and nodular exocrine hyperplasia; suppurative and interstitial pneumonia	Spontaneous
7	Leopard (Panthera pardus)	F	9.8	Good	- / -	*	IN, renal intratubular concrements, renal, pulmonary and meningeal mineralization, renal amyloidosis; parathyroid gland: adenoma; exocrine pancreas: nodular hyperplasia; suppurative endometritis; ulcerative gastritis	Spontaneous
8	Leopard (Panthera pardus)	M	16	Cachectic	- / -	< 50	GN, IN; peritoneal cavity: neuroendocrine tumor (suspected islet cell tumor);	Spontaneous
9	Leopard (Panthera pardus)	F	13	Cachectic	- / -	< 50	Lumbar vertebra: osteosarcoma; mammary gland: simple carcinoma; adrenal gland: cortical hyperplasia	Euthanasia
10	Leopard (Panthera pardus)	M	15	Good	- / -	< 50	IN, pyelitis, TD; osteomyelitis; discospondylitis; cardiac lipomatosis	Spontaneous
11	Leopard (Panthera pardus)	M	7	Good	n.d.	n.d.	Asphyxia due to airway obstruction by meat	Spontaneous
12	Leopard (Panthera pardus)	F	3	Good	- / -	n.d.	Pyelitis, TD; disseminated hylaline microthrombi; myocardial degeneration; hemorrhagic gastritis; cardiac lipomatosis	Spontaneous
13	Leopard (Panthera pardus)	F	17	Moderate	n.d.	n.d.	IN, renal intratubular concrements; uterus, ovary: leiomyoma; pancreas: islet cell tumor	Spontaneous
14	Lion (Panthera leo)	F (n)	13	Good	+ / -	70	IN, TD; spleen: hemangiosarcoma; liver: carcinoma; pyometra; granulomatous pneumonia; lymphohistiocytic gastritis	Spontaneous
15	Lion (Panthera leo)	F	12	Obese	+ / -	n.d.	Ovary: leiomyoma; chronic lymphohistiocytic pododermatitis	Spontaneous
16	Lion (Panthera leo)	F	3	Good	- / -	250	GN, IN, pyelitis, TD, renal intratubular concrements; tongue: papilloma; ulcerative gastritis; enteritis^a^; fibrino-purulent to necrotizing and interstitial pneumonia; cardiac lipomatosis	Spontaneous
17	Lion (Panthera leo)	F	11	Good	+ / -	*	IN, TD, renal intratubular concrements; pyometra; catarrhal enteritis with crypt loss, re-epithelialization and crypt abscesses; DJD	Spontaneous
18	Lion (Panthera leo)	F	6	Good	- / -	160	IN, metastatic suppurative nephritis, renal intratubular concrements; erosive-ulcerative gastritis; catarrhal enteritis with crypt regeneration, crypt abscesses, fibrosis, villus stunting and villus fusion; granulomatous pneumonia	Spontaneous
19	Lion (Panthera leo)	M	18	Good	- / -	200	GN, IN, pyelitis, TD, renal intratubular concrements; enteritis^a^; suppurative pneumonia; hepatic and renal cysts; exocrine pancreas: nodular hyperplasia; DJD; heel: ulcerative dermatitis	Euthanasia
20	Lion (Panthera leo)	M (n)	5	Good	+ / -	> 300	GN, IN, metastatic suppurative nephritis, renal intratubular concrements; amyloidosis of exocrine pancreas and thyroid gland; enteritis^a^	Euthanasia
21	Tiger (Panthera tigris)	M	16	Moderate	- / -	270	IN, TD, renal intratubular concrements; pleura: mesothelioma; pancreas: carcinoma; fibrino-purulent to necrotizing pneumonia; hepatitis; ulcerative gastritis	Spontaneous
22	Tiger (Panthera tigris)	F	22	Moderate	- / -	> 300	GN, pyelitis, renal intratubular concrements; uterus: leiomyoma; thyroid gland: unilateral adenoma, focal unilateral hyperplasia; cortical hyperplasia of adrenal gland	Euthanasia
23	Tiger (Panthera tigris)	F	15	Good	n.d.	220	IN, TD, renal intratubular concrements; uterus: leiomyoma; interstitial pneumonia; traumatic pneumothorax, multifocal pulmonary mineralization	Spontaneous
24	Tiger (Panthera tigris)	F	8	Good	n.d.	100	Tongue: papilloma; lymphohistiocytic endometritis with cystic glandular hyperplasia; interstitial pneumonia	Euthanasia
25	Tiger (Panthera tigris)	M	19	Good	- / -	< 50	GN, IN, TD, renal mineralization; pleura: mesothelioma; thyroid gland: adenoma; parathyroid hyperplasia; mineralization (vessels of hippocampus, lung); DJD; interstitial pneumonia; lymphohistiocytic enteritis	Spontaneous
26	Tiger (Panthera tigris)	F (n)	19	Good	- / -	170	GN, IN, pyelitis, TD, renal intratubular concrements, renal mineralization; thyroid gland: adenoma; several organs^b^: myeloma	Spontaneous
27	Tiger (Panthera tigris)	M	19	Moderate	- / -	n.d.	GN, IN, pyelitis, TD, renal intratubular concrements; lung: bronchioloalveolar carcinoma; adrenal gland: unilateral pheochromocytoma and bilateral, cortical hyperplasia; parathyroid hyperplasia; hyperplasia of exocrine pancreas; eosinophilic enteritis	Spontaneous
28	Tiger (Panthera tigris)	F	18	Moderate	n.d.	30	GN, IN, pyelitis, TD; uterus: leiomyoma; suppurative and interstitial pneumonia; cardiac lipomatosis	Spontaneous
29	Tiger (Panthera tigris)	F	22	Good	n.d.	175	GN, IN, pyelitis, TD, renal intratubular concrements; uterus: leiomyoma; thyroid gland: multiple adenomas; nodular cortical hyperplasia of adrenal gland; brain: leukoencephalopathy with cerebral leukomalacia, gliosis, gemistocytes, spheroids and internal hydrocephalus; cardiac lipomatosis; DJD	Euthanasia
30	Tiger (Panthera tigris)	F	18	Obese	- / -	n.d.	IN, pyelitis, TD, renal intratubular concrements, renal mineralization; cardiac lipomatosis; nodular cortical hyperplasia of adrenal gland; interstitial pneumonia	Spontaneous
31	Tiger (Panthera tigris)	F	6	Good	- / -	n.d.	IN, pyelitis, metastatic suppurative nephritis, renal intratubular concrements; DJD; lymphoplasmacytic gastritis; enteritis^a^; cardiac lipomatosis	Euthanasia
32	Tiger (Panthera tigris)	F	19	Good	- / -	70	IN, pyelitis, TD, renal intratubular concrements, renal mineralization; DJD; cortical hyperplasia of adrenal gland; lymphocytic gastritis; cardiac lipomatosis	Spontaneous
33	Tiger (Panthera tigris)	F	1	Good	- / -	300	GN, IN, TD, renal intratubular concrements; systemic mineralization (including kidney); myocardial degeneration	Spontaneous
34	Tiger (Panthera tigris)	M	0.8	Moderate	- / -	150	IN, TD, renal intratubular concrements; catarrhal enteritis^c^; cardiac lipomatosis	Spontaneous
35	Tiger (Panthera tigris)	F	1	Poor	- / -	110	Renal intratubular concrements, systemic mineralization (including kidney); thigh: ulcerative dermatitis; catarrhal enteritis	Spontaneous
36	Tiger (Panthera tigris)	M	1	Cachectic	- / -	200	Renal intratubular concrements; systemic mineralization (including kidney); ulcerative glossitis; enteritis^a^	Spontaneous
37	Tiger (Panthera tigris)	F	3	Good	n.d.	180	IN, pyelitis, TD, renal intratubular concrements, metastatic suppurative nephritis, renal mineralization; enteritis^a^; panmyelophthisis; erosive gastritis	Spontaneous
38	Tiger (Panthera tigris)	M	0.5	Good	- / -	< 50	TD; limb malformations, thymus atrophy	Euthanasia

- = negative; + = positive; DJD = degenerative joint disease; F = female; FeLV = feline leukemia virus; FIV = feline immunodeficiency virus; GN = glomerulonephritis; IN = interstitial nephritis; M = male; n = neutered; n.d. = not determined; TD = tubular degeneration; Urea = urea concentration in fluid of anterior eye chamber (reference range < 50 mg/dl). * = urea nitrogen content was evaluated, but the value was excluded from the study due to an interval longer than 48 hours between death and necropsy.

^a^Catarrhal enteritis resembling parvovirus infection morphologically (crypt dilation with accumulation of cellular debris, villous fusion and atrophy, and crypt regeneration).

^b^Bone marrow, lymph node, spleen, pituitary gland.

^c^Enteritis due to infection with *Salmonella typhimurium* and *Clostridium perfringens* type A as detected by microbial investigation.

### Histopathology

Whole body necropsies were performed by different pathologists. Samples for histopathology taken from the majority of animals included: respiratory and gastrointestinal tract, liver, pancreas, urogenital tract, heart, hematopoietic organs, musculoskeletal system, endocrine organs, peripheral as well as central nervous system. Samples were fixed in 10% neutral buffered formalin, dehydrated and embedded in paraffin wax. Sections of 3–50020μm thickness were mounted on glass slides (Engelbrecht Medizin- und Labortechnik GmbH, Edermünde, Germany) and stained with hematoxylin and eosin (H&E). Applied histopathological stainings were performed according to standard procedures as described elsewhere [30]. All kidney sections were stained with Periodic acid-Schiff (PAS) reaction, Congo-red, azan and May-Grünwald’s trichrome staining to investigate basement membranes, amyloid depositions and the extracellular matrix, respectively. Congo-red and Campbell-Switzer silver impregnation were used to detect amyloid deposition or neurofibrillary tangles in all brain specimens, respectively. Congo-red was also applied to identify amyloid deposition in pancreatic islets in all animals. Warthin-Starry silver impregnation was used to detect *Helicobacter*-like organisms in all stomach samples. In addition, the following special stainings were only applied to subsets of cases. In these cases, findings in the H&E-staining require verification: Fouchet staining for hepatic cholestasis, Grocott silver impregnation for fungal organisms, von Kossa’s staining for calcium and phosphorus deposition, Turnbull’s blue staining for iron depositions (hemosiderin), and Gram, Ziehl-Neelsen staining as well as the PAS reaction for possible organisms in the lung of animals with pneumonia.

### Immunohistochemistry

Immunohistochemistry was performed on formalin-fixed and paraffin embedded samples according to standard procedures. Briefly, antigen retrieval was performed in citrate buffer (pH 6.0, 20 min, 95°C) followed by blocking of endogenous peroxidase activity with 0.5% hydrogen peroxide in 70% ethanol for 30 min. Subsequent to the application of normal goat serum (20% in phosphate buffered saline, 30 min), primary antibodies were added and incubated for approximately 2 hours followed by application of secondary antibodies for 30 min. The avidin-biotin complex (ABC) method (VECTASTAINING Elite ABC Kit, Vector Laboratories, Burlingame, California) was performed according to the manufacturer instructions and 3, 3′-diaminobenzidine-tetrahydrochloride (DAB, Sigma-Aldrich, Munich, Germany) was used as chromogen. Sections were counterstained with Mayer’s haematoxylin. Primary antibodies directed against parvovirus antigen (monoclonal mouse anti-parvovirus antibody, dilution 1:500, clone CPV1-2A1, Custom Monoclonals International, Sacramento, California) and FeLV antigen (monoclonal mouse anti-FeLV antibody, dilution 1:200, clone C11D8, Custom Monoclonals International) were used for intestinal samples of animals with enteritis (n = 13). Antibodies directed against pan-morbillivirus antigen (polyclonal rabbit anti-canine distemper nucleoprotein antibody, #25, dilution 1:2000, kindly provided by Professor C. Örvell, Central Microbiological Laboratory of Stockholm county, council Stockholm, Sweden; [31]) were applied for the detection of viral antigen in renal samples of all animals. Astrocytes in the brain were visualized by using an anti-glial fibrillary acidic protein antibody (GFAP; polyclonal rabbit anti-cow GFAP antibody, dilution 1:2000, Dako, Hamburg, Germany) in animal no. 29. If appropriate, primary antibodies directed against CD3 for T cells (polyclonal rabbit anti-CD3 antibody, dilution 1:2000, Dako) and antibodies specific for synaptophysin for neuroendocrine cells (monoclonal mouse anti-human synaptophysin, dilution 1:100, Dako, clone DAK-SYNAP) were used in few cases to verify the cellular origin of neoplastic lesions. Secondary, biotinylated antibodies included goat anti-mouse IgG (Vector Laboratories, Burlingame, California), and goat anti-rabbit IgG (Vector Laboratories), respectively.

### Additional diagnostic tests

Fluid of the anterior eye chamber was collected (30/38 animals) to evaluate the urea concentration (Urea test strips, Reference number 131299990350, DiaSys Diagnostic Systems GmbH, Holzheim, Germany). The amount of blue discoloration of the test strip by ammonium [mm] is correlated with the urea concentration [mg/ml] in the eye chamber. Results were estimated according to the values provided by the manufacturer. Postmortem urea concentration in the eye chamber correlates with blood urea concentration ante-mortem (reference value < 50 mg/dl) [32]. No data regarding the urea nitrogen stability in the eye chamber after death exist in large felids, however based on canine, equine and bovine data it can be assumed, that the value is stable at least for 36 hours at moderate and lower temperatures [33–35]. In one study, it was shown that the urea nitrogen value increased severely after 48 hours [34]. To avoid false positive results, urea nitrogen investigations were only included in the study, if the time between death and necropsy was less than 48 hours. Therefore, the urea nitrogen value of 27 out of 30 large felids was included in the study only. However, it should be considered that the reference value is validated for humans, domestic cats, dogs and cattle but has not been validated for wild felids so far. Blood was collected from 31 of 38 animals and submitted to the small animal clinic (University of Veterinary Medicine Hannover) for the detection of FIV antibodies and FeLV antigen using the SNAP Combo FIVAb ⁄ FeLVAg Test (Idexx Laboratories, Ludwigsburg, Germany). In one tiger with a catarrhal enteritis (animal no. 34), unfixed frozen intestinal tissue samples were submitted to the Department of Microbiology for bacteriological investigation in accordance with the owners’ decision.

### Statistical analysis

Statistical analysis was performed using R version 3.1.2 [36]. Age of animals and urea concentration in the anterior eye chamber were tested for normal distribution by evaluating histograms, Q-Q plots and applying Shapiro-Wilk test. As the age was not normally distributed (p < 0.01), median, minimal and maximal values were given in the manuscript.

The impact of age, sex and species on the presence of pathologic findings was assessed in multifactorial logistic regression analyses. Significance was tested by the likelihood ratio test. For p < 0.05, the odds ratio (OR) is given for age increased by 1 or changes in sex (e.g. male compared to female), respectively. For impact of species, significant likelihood ratio tests were followed by pairwise comparison of distinct species using the Wald test and the OR is given for p < 0.05. The impact of FIV infection on the frequency of pathologic lesions was assessed similarly. It has to be considered that differences in lifespan between individual felid species may have an impact on the development of various lesions, however this need to be analyzed in a larger cohort.

Cohen’s kappa statistics followed by testing the null-hypothesis that the estimated kappa is related to chance, as well as the Bangdiwala's agreement chart were used to determine agreement between several findings. This procedure was also applied to test for association of several findings with an increased (> 50 mg/dl) urea concentration in the anterior eye chamber. For p < 0.05, judgement of the estimated kappa was made as follows: if kappa is between 0 and 0.2, "slight agreement", if 0.2–0.4, "fair agreement", if 0.4–0.6, "moderate agreement", if 0.6–0.8, "substantial agreement", if 0.8–1.0, "almost perfect agreement" [37].

## Results

Detailed information about investigated animals including species, gender, age, nutritional status, detection of FeLV and FIV, urea concentration measured in the anterior eye chamber, significant pathologic findings and manner of death is given in Table 1. A comprehensive overview of all degenerative, inflammatory and neoplastic changes in each animal included in the study is given in S1 Table.

### Urinary system

#### Inflammation and degenerative lesions

**Fig 1:**
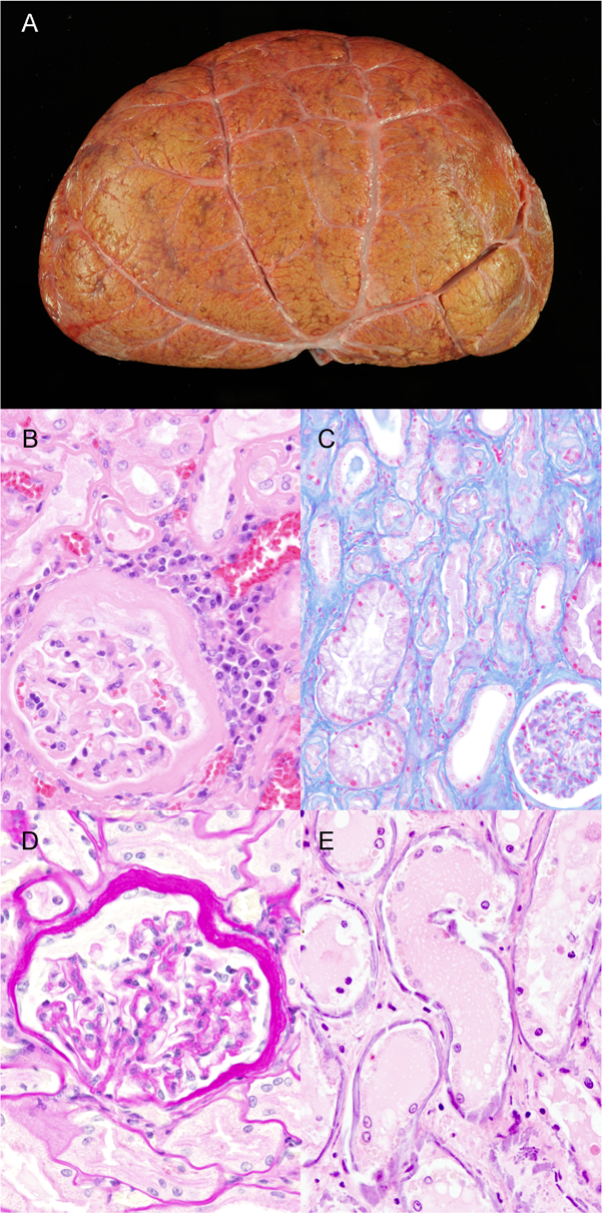
Renal lesions in wild felids A) Kidney, tiger, 19 years, male (animal no. 27). Chronic interstitial nephritis with an irregular surface of the kidney. B) Kidney, cougar, 18 years, female (animal no. 4). Membranous glomerulonephritis with moderate to severe thickening of Bowman’s capsule. Interstitial infiltrations consisting of lymphocytes, plasma cells and fewer macrophages. H&E-staining. C) Kidney, lion, 6 years, female (animal no. 18). Moderate diffuse interstitial fibrosis. Azan staining. D) Kidney, cougar, 18 years, female (animal no. 4). Membranous glomerulonephritis and a moderate, diffuse thickening of Bowman’s capsule are present. Periodic Acid-Schiff reaction. E) Kidney, tiger, 1 year, female (animal no. 33). Tubular basement membranes displaying severe diffuse depositions of basophilic, plaque-like extracellular material (mineralization). H&E-staining.

In the present study 33 out of 38 animals (87%) showed renal lesions (Table 2, S1 Table) without any sex predisposition. They were classified as tubular, interstitial, glomerular pathology and miscellaneous. There was a substantial agreement between the occurrence of interstitial and tubular changes (kappa = 0.68, p < 0.0015), as well as a moderate agreement between the presence of interstitial and glomerular findings (kappa = 0.40, p < 0.0045). However, the association between glomerular changes and tubular changes was not significant (kappa = 0.21, p = 0.084). The risk for the presence of interstitial as well as glomerular changes significantly increased with age of the animals (OR = 1.17, p = 0.0092 and OR = 1.14, p = 0.0135, respectively). Tubular changes included luminal concrement accumulations (23/38 animals; 61%), tubular epithelial degeneration and necrosis comprising luminal cellular detritus and/or cytoplasmic storage of hyaline/lipid droplets (21/38 animals; 55%), as well as proteinaceous casts (19/38 animals; 50%). Tigers revealed a higher risk for the occurrence of tubular degeneration and necrosis compared to leopards (OR = 7.8, p = 0.0345). By statistical analysis, the frequency of proteinaceous casts increased with age (OR = 1.11, p = 0.0396) and was higher in lions compared to leopards (OR = 17.5, p = 0.035). Interstitial kidney lesions consisted of lympho-plasmacytic interstitial nephritis (27/38 animals; 72%; Fig 1A and 1B), fibrosis (26/38 animals; 68%, Fig 1C), eosinophilic interstitial nephritis (4/38; 11%) and metastatic-purulent nephritis (4/38; 11%). There was moderate agreement between the occurrence of increased urea concentration in the anterior eye chamber and concrement accumulation in tubular lumina (kappa = 0.59, p < 0.003) as well as fair agreement with dilated tubuli (kappa = 0.29, p < 0.042). However, other renal lesions, gastritis or enteritis were not significantly associated with increase in urea concentration in the anterior eye chamber. Interstitial nephritis occurred in animals with a median age of 13 years (ranging from 0.5 to 22 years) with an increasing frequency with age (OR = 1.14, p < 0.016) and there was a significant substantial agreement with interstitial fibrosis (kappa = 0.69, p < 0.0002) and fair agreement with glomerular sclerosis (kappa = 0.35, p < 0.0066). There was also an association between age and the presence of interstitial fibrosis (OR = 1.15, p < 0.011). Five animals with interstitial nephritis (5/27 animals; 19%) displayed an erosive to ulcerative gastritis, however there was no statistical significant agreement between the occurrence of these findings (kappa = 0.17, p = 0.1048). Morbillivirus antigen was not detected immunohistochemically in kidneys of captive felids. The frequency of eosinophilic interstitial nephritis decreased with age (OR = 0.80, p = 0.016). Glomerular lesions included glomerulonephritis (GN; 16/38 animals; 42%), glomerulosclerosis (12/38 animals; 32%) and amyloidosis (1/38 animals; 3%). GN was classified as membranoproliferative (7/38 animals; 18%), mesangioproliferative (5/38 animals; 13%) and membranous (4/38 animals; 11%; Fig 1B and 1D). The frequency of GN increased with age (OR = 1.14, p = 0.014). However, the occurrence of GN was not significantly associated with interstitial nephritis. Additional lesions included loss of nephrons (16/38 animals; 42%), lympho-plasmacytic pyelitis (15/26 animals; 57%; renal pelvis was only available in 26 animals) and cytoplasmic storage of a yellow pigment within tubular epithelial cells (11/38 animals; 29%). The frequency of both loss of nephrons and lympho-plasmacytic pyelitis raised with increasing age (OR = 1.15, p < 0.01 and OR = 1.13, p < 0.036, respectively). Pyelitis was further associated with the presence of glomerular changes (kappa = 0.46, p < 0.011). Mineralization of basement membranes was present in 4 animals that did not show systemic mineralization (11%). Polycystic lesions in kidney and liver resembling polycystic kidney disease were observed in one lion (animal no. 19; [38]).

**Table 2:** Degenerative and inflammatory kidney lesions in 38 non-domestic felids.

		Affected animals (percent)
**Tubular lesions**		31 (82%)
	Intra-tubular concrement	23 (61%)
	Tubular degeneration and necrosis with intra-luminal cellular detritus and cytoplasmic storage of hyaline or lipid-droplets	21 (55%)
	Proteinaceous casts	19 (50%)
	Dilated tubuli	12 (32%)
**Interstitial lesions**		29 (76%)
	Lympho-plasmacytic inflammation	27 (72%)
	Interstitial fibrosis	26 (68%)
	Metastatic-purulent inflammation	4 (11%)
	Eosinophilic inflammation	4 (11%)
**Glomerular lesions**		17 (45%)
	Membranoproliferative GN	7 (18%)
	Mesangioproliferative GN	5 (13%)
	Membranous GN	4 (11%)
	Glomerulosclerosis	12 (32%)
	Amyloidosis	1 (3%)

GN = glomerulonephritis. Lesions listed were present in several animals simultaneously.

#### Metastatic mineralization

**Fig 2:**
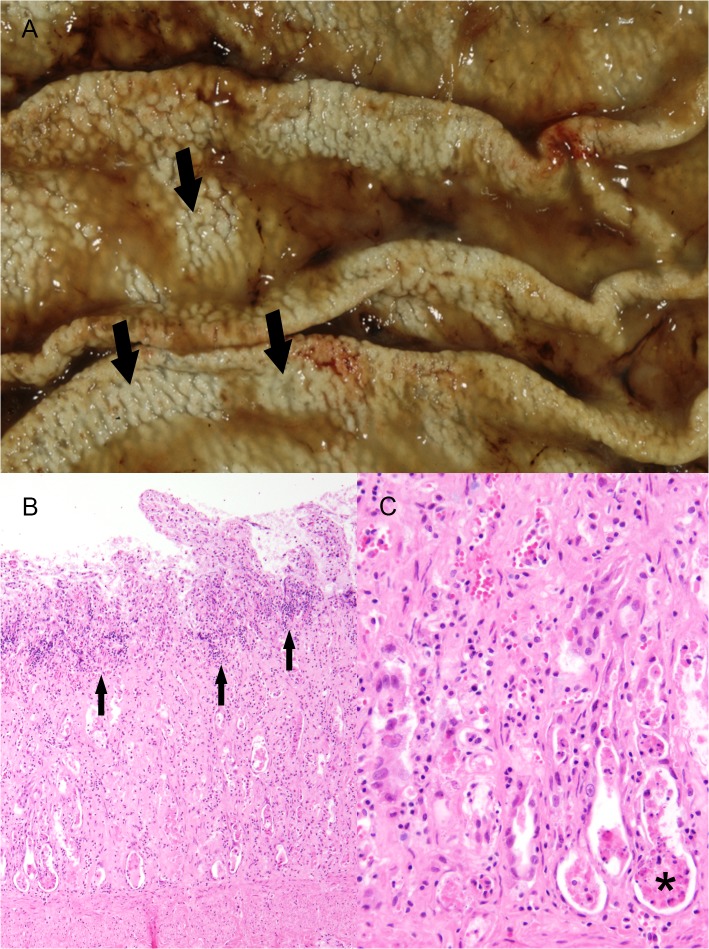
Gastrointestinal lesions in wild felids. A) Stomach, tiger, 1 year, male (animal no. 36). Severe diffuse mineralization of the gastric mucosa consisting of white, granular, crunchy material (arrows). B) Jejunum, tiger, 3 years, female (animal no. 37). Villus fusion, shortening and loss of epithelial cells with cellular debris and multifocal, moderate, lympho-plasma-histiocytic inflammation (arrows). H&E-staining. C) Jejunum, tiger, 3 years, female (animal no. 37). Higher magnification of Fig 2B, jejunum with crypt dilatation and accumulation of cellular debris in the crypts (asterisk). H&E-staining.

Three 11- and 12-month-old tigers (animal nos. 33, 35 and 36) from the same zoological garden died within a few months. In these animals kidneys displayed bilaterally a coarse granular surface and a narrow (0.5–0.8 cm) renal cortex. Myocardium, blood vessels, lung, stomach, duodenum and/or thyroid gland and choroid plexus displayed multifocal, pinpoint, white, partly calcified areas. Animals exhibited an increased urea concentration in the anterior eye chamber of 300, 110 and 200 mg/dl, respectively. One animal (animal no. 36) showed an ulcerative glossitis located at the ventro-lateral aspect of the tongue. Bone mineralization and consistency were unremarkable at gross examination and there were no indications of fibrous osteodystrophy. Histopathologically, multifocal mineralization of basement membranes and/or elastic fibers of myocardium, kidney (Fig 1E), lung, aorta, trachea, as well as gastric (Fig 2A) and duodenal mucosa was detected. The kidneys of one animal (animal no. 33) displayed a focal, interstitial nephritis characterized by severe infiltration of eosinophils and moderate numbers of lymphocytes and plasma cells accompanied by mild interstitial fibrosis, membranoproliferative glomerulonephritis and mild tubular degeneration with dilated tubuli, proteinaceous casts, intratubular concrements and intraepithelial yellow pigment deposition. In all affected animals parathyroid glands were without significant findings.

A 19-year-old male tiger (animal no. 25) and a 9-year-old female leopard (animal no. 7) with interstitial nephritis revealed similar mineralization of kidneys, lung, meninges and blood vessels of hippocampus associated with parathyroid hyperplasia or adenoma, respectively.

### Alimentary tract

#### Inflammation

Microscopic evaluation of the stomach revealed mucosal inflammation in 10 of 38 animals (26%) that was not associated with age or animal species but more common in female individuals (OR = 7.80, p = 0.0278). These included erosive to ulcerative (5/38; 13%), lympho-plasma-histiocytic (3/38; 8%) and hemorrhagic gastritis (2/38; 5%). In contrast to non-purulent and hemorrhagic inflammation, erosive to ulcerative gastritis coexisted with interstitial nephritis. In one case, ulcerative gastritis, azotemia and interstitial nephritis was associated with a parathyroid adenoma in one leopard (animal number 7). Warthin-Starry silver impregnation demonstrated *Helicobacter*-like organisms in the gastric mucosa of three animals including 1 cheetah (animal no. 1), 1 cougar (animal no. 5) and 1 tiger (animal no. 26) and the presence of spirochetes was not associated with concurrent gastric inflammation.

Thirteen animals (34%) revealed enteritis in the small intestine with an age ranging from 0.7 to 19 years (median: 6 years). The risk for the occurrence of enteritis significantly decreased with age (OR = 0.89, p = 0.0301) but was not influenced by gender or species. Enteritis was not associated with increased urea concentration in the anterior eye chamber. In 6 of 13 felids (3 lions, 2 tigers, 1 cheetah), catarrhal enteritis characterized by crypt dilation with luminal accumulation of cellular debris, villous atrophy, thickening of the villi (villous fusion) and re-epithelialization (crypt regeneration) was noticed (parvovirus-like enteritis; Fig 2B and 2C). One of these animals (1 tiger, animal no. 37) displayed a panmyelophthisis. However, FeLV- and parvovirus-antigen were not detected in any case. Enteritis was associated with FIV infection in 2 lions (animal no. 17: catarrhal enteritis with crypt abscesses, crypt loss and re-epithelialization; animal no. 20: parvovirus-like enteritis) as determined by detectable antibodies in blood. *Salmonella typhimurium* and *Clostridium perfringens* type A were identified in intestinal samples (microbiological examination) from an 8-month-old male tiger with catarrhal enteritis. Eosinophilic inflammation within the intestinal mucosa without evidence for parasitic infection was present in a 19-year-old male tiger with moderate nutritional condition.

#### Neoplasms

The risk for the general presence of neoplasia increased with age (OR = 1.23, p = 0.0003) but was not influenced by sex or species. In the alimentary tract, tumors were detected in the tongue only (papilloma; 2/38 animals; 5%).

### Liver and pancreas

#### Hyperplasia and neoplasms

Similar to neoplasia, the general presence of hyperplastic lesions increased with age (OR = 1.42, p < 0.0001) but was independent from gender or species. There was no significant association between the occurrence of hyperplasia and neoplasia (kappa = 0.21, p = 0.0972). The exocrine pancreas was assessed in 33 felids and nodular hyperplasia was present in 5 animals (5/33 animals; 15%). Male compared to female felids showed a higher frequency of hyperplastic changes in the pancreas (OR = 9.20, p = 0.0334). Neoplasms found in liver and pancreas included a single hepatic (animal no. 14; 1/38 animals; 3%) and pancreatic carcinoma (animal no. 21; 1/38 animals; 3%) in each animal.

### Endocrine organs

#### Degenerative lesions

No degenerative lesions including amyloid deposition were identified in the pancreas.

#### Hyperplasia and neoplasm

Eleven of 16 hyperplastic lesions detected in 12 of 38 animals were located in endocrine organs including moderate nodular hyperplasia of adrenal cortex (6/12 animals; 50%) as well as moderate hyperplasia of parathyroid (4/12 animals; 34%) and thyroid glands (1/12 animals; 8%). Both adrenal and parathyroid hyperplasia were more common in older animals (OR = 1.61, p = 0.0004 and OR = 1.26, p = 0.0335, respectively).

**Fig 3:**
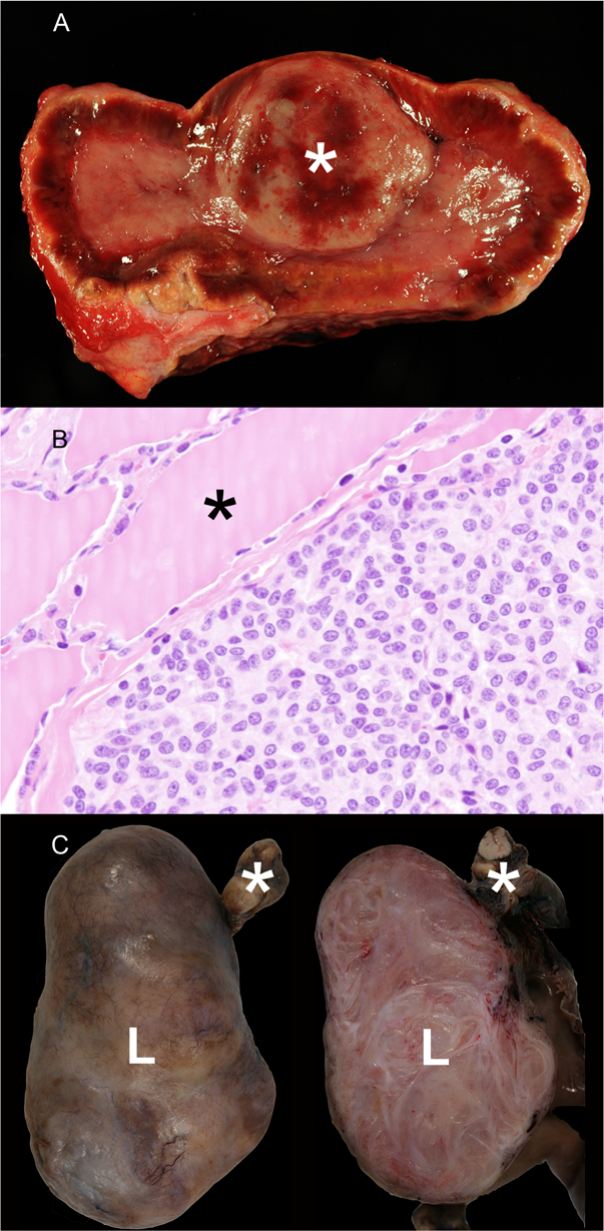
Endocrine and genital neoplasia in wild felids. A) Adrenal gland, tiger, 19 years, male (animal no 27). Unilateral pheochromocytoma (asterisk). B) Parathyroid gland, leopard, 9 years, female (animal no. 7). Parathyroid gland adenoma characterized by a solid growth pattern. The parathyroid gland adenoma displayed a capsule and compresses adjacent normal follicles of the thyroid gland (asterisk). H&E-staining. C) Ovary, leopard, 17 years, female (animal no. 13). The left part of the picture shows an encapsulated, well demarcated leiomyoma (L) attached to normal ovary tissue (asterisk). The right part of the picture demonstrates the firm, nodular cut surface of the leiomyoma (L) that is characterized by irregularly arranged interwoven tissue bundles.

Endocrine organs were most commonly affected by neoplasms (11/34 tumors; 32%). Thyroid tumors including adenomas (n = 5) and a carcinoma (n = 1) represented the second most common neoplasms detected in captive wild felids (6/34 tumors; 18%; age ranged from 18 to 22 years with median age of 19 years) and the risk was significantly increasing with age (OR = 3.65, p < 0.0001). Affected animals were in a poor (n = 1), moderate (n = 1) or good (n = 4) body condition. A pancreatic islet cell tumor was present in a female leopard (animal no. 13), and there was a neuroendocrine tumor in the abdominal cavity of a male leopard (animal no. 8) that was assumed to be a pancreatic islet cell tumor, too (n = 2). A pituitary carcinoma (n = 1) and a pheochromocytoma (n = 1; Fig 3A) were present. Parathyroid hyperplasia (animal no. 25) or adenoma (animal no. 7; Fig 3B) were found in 2 animals. In addition, these animals showed an associated interstitial nephritis. In these cases metastatic mineralization was present in kidneys, lung, meninges and blood vessels of the hippocampus while the bones appeared unremarkable (see chapter metastatic mineralization).

### Reproductive system

#### Inflammation

Within the uterus, inflammatory lesions were found in 4 of 24 felids (17%) most commonly characterized by purulent endometritis and/or pyometra (n = 3). Lympho-histiocytic endometritis was associated with cystic glandular hyperplasia in one animal. The presence of endometritis was not influenced by age or animal species.

#### Neoplasms

Organs of the reproductive tract were second most commonly affected by neoplasms (8/34 tumors; 24%). Tumors included uterine (4 tigers, 1 leopard) and ovarian (1 lion, 1 leopard; Fig 3C) leiomyomas, which represented the most commonly detected tumor type in the present study (7/34 tumors; 21%; age ranged from 12 to 22 years with median age of 18 years). The occurrence of leiomyomas increased with age (OR = 1.30, p = 0.0053). A simple carcinoma of the mammary gland was present in a 13-year-old female leopard (animal no. 9).

### Central nervous system

#### Leukoencephalopathy

**Fig 4:**
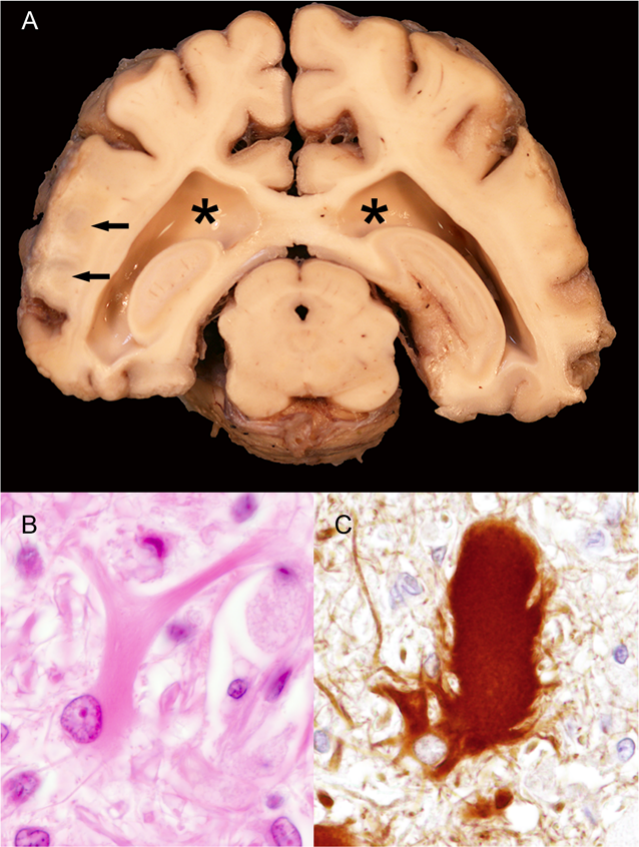
Pathologic features of large felid leukoencephalomyelopathy. A) Cerebrum, tiger, 22 years, female (animal no 29). Large felid leukoencephalomyelopathy characterized by bilateral dilatation of the lateral ventricles (asterisks) and malacic foci within the white matter (arrows). B) Cerebrum, white matter, tiger, 22 years, female (animal no 29). Gemistocytic astrocyte with prominent cytoplasmic processes and abundant homogenous eosinophilic cytoplasm. C) Cerebrum, white matter, tiger, 22 years (animal no 29), female. Gemistocytic astrocyte with a cytoplasmic positive GFAP (glial fibrillary acidic protein)-reaction. GFAP, immunohistochemistry, ABC, DAB-method.

A 22-year-old female tiger (animal no. 29) presented with not further specified “chronic complaints of old age” and the referring veterinarian diagnosed a chronic renal failure based on clinical pathological investigations. However, it was not possible to determine the onset of the clinical signs. Additionally, degenerative joint disease due to reduced movement was suspected and finally the animal was recumbent leading to euthanasia. In this animal degenerative changes of the white matter restricted to the brain were associated with a mild, bilateral, internal hydrocephalus *ex vacuo*. The cerebrum revealed a subventricular white matter malacia and dilatation of the lateral ventricles (Fig 4A). Histology showed a diffuse astrogliosis with numerous gemistocytes (Fig 4B and 4C) in this area, which was confirmed by GFAP immunohistochemistry. In addition few spheroids were found in the brain stem of this animal.

#### Miscellaneous

Neuronal lipofuscinosis (12/38 animals; 32%), meningeal fibrosis (10/38 animals; 26%), dilated myelin sheaths, myelinophagia and spheroids (6/38 animals; 16%), mineralization of the choroid plexus, meninx, and meningeal vessels independent from systemic mineralization (5/38 animals; 13%) as well as lymphocytic/lympho-histiocytic meningitis (3/38 animals; 8%) were found. Neuronal lipofuscinosis was significantly associated with age (OR = 1.14, p = 0.0229). Male felids were significantly predisposed for the presence of meningeal fibrosis compared to females (OR = 12.22, p = 0.0119). Amyloid depositions and neurofibrillary tangles were not detected in the cerebrum of captive wild felids.

#### Neoplasms

In an 18-year-old female cougar (animal no. 4) with a history of neurological signs, a psammomatous meningioma was located at the cerebral meninges of the cranium. This neoplasm was associated with mild multifocal lympho-histiocytic meningitis, but there was no compression of the adjacent brain parenchyma.

### Respiratory tract and body cavities

#### Inflammation

Pneumonia was evident in 12 felids including 6 tigers, 4 lions, 1 leopard and 1 cougar (age ranged from 3 to 19 years). Histologically, either interstitial (n = 4), purulent (n = 2), granulomatous (n = 2) or fibrinous to necrotizing (n = 1) pneumonia, or a combination of interstitial and purulent (n = 2), as well as interstitial and fibrinous inflammation (n = 1) were detected. The fibrinous pneumonia of 1 lion (animal no. 16) showed intralesional fungal hyphae measuring approximately 6 μm in width with thin parallel walls, regular septa and dichotomous acute angular branching suggestive of *Aspergillus* sp. In one lion with granulomatous pneumonia FIV-specific antibodies were detected. In the remaining animals, no etiology was identified based on gross findings, histology and histochemistry including Gram, Ziehl-Neelsen staining and PAS reaction. However, no additional tests (e.g. microbiological or virological examination) were carried out. Additional findings observed in the lungs comprised anthracosis (10/38 animals; 26%), interstitial and pleural fibrosis (8/38 animals; 21%), mild multifocal mineralization of vessels and alveolar septa not associated with systemic mineralization (animal nos. 5, 8, 23, 26, 27 and 32; 6/38 animals; 16%) and hemosiderosis (5/38 animals; 13%). The risk for presence of pulmonary anthracosis as well as interstitial and pleural fibrosis significantly increased with age (OR = 1.20, p = 0.0046 and OR = 1.20, p = 0.0096, respectively). The presence of pneumonia was not associated with the occurrence of anthracosis, interstitial and pleural fibrosis, pulmonary mineralization and hemosiderosis, and there was no sex or species predisposition for the presence of any respiratory changes.

#### Neoplasms

**Fig 5:**
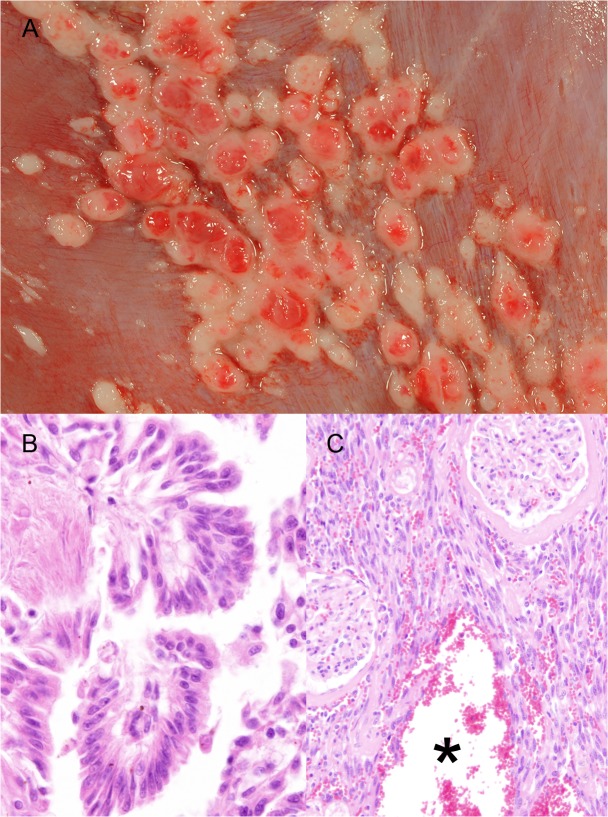
Pleural and vascular tumors in wild felids. A) Pleura costalis, tiger, 19 years, male (animal no 25). Mesothelioma of the diaphragmatic pleura characterized by numerous elevated grayish-red confluent nodules. B) Pleura, cheetah, 10 years, male (animal no 1). Epithelial component of a mesothelioma displaying a papillary growth pattern. H&E-staining. C) Kidney, cougar, 18 years, female (animal no 4). A hemangiosarcoma characterized by a large blood filled cavity (asterisk). H&E-staining.

In the lung of a 19-year-old male tiger (animal no. 27), a bronchioloalveolar carcinoma was detected. Pleural mesotheliomas (Fig 5A and 5B) were observed in 3 animals including 1 cheetah (animal no. 1) and 2 tigers (animal nos. 21 and 25), all of which were male felids.

### Lymphoreticular and hematopoietic organs

#### Retroviral infection

FIV antibodies were identified in the blood of 3 female and 1 male lion (4/38 animals; 11%; animal nos. 14, 15, 17 and 20), while FeLV antigen was not present in any investigated sample. In all FIV-infected animals, various inflammatory lesions were detected including catarrhal enteritis (n = 2; see inflammation of alimentary tract), pyometra (n = 2, see inflammation of genital tract), granulomatous pneumonia (n = 1, see inflammation of respiratory tract) and metastatic purulent nephritis (n = 1, see inflammation of urinary system). Furthermore, 2 FIV-positive lions revealed neoplastic lesions comprising a hemangiosarcoma and a hepatic carcinoma (animal no. 14), as well as an ovarian leiomyoma (animal no. 15). Logistic regression analysis revealed that FIV infected felids have a higher risk for the development of endometritis (OR = 16, p = 0.0282), whereas the risk for enteritis, pneumonia, metastatic purulent nephritis or neoplasia was not influenced by FIV infection.

#### Miscellaneous

Lymphoid depletion of the spleen, pulmonary, mesenteric, iliac lymph nodes and/or Peyer’s patches, partly associated with necrosis, was found in 11 female felids (11/38 animals; 29%). These lesions were not associated with FIV infection and their risk was not influenced by age or species. However, lymphoid depletion was more frequently detected in female felids (OR = 9.29, p = 0.0150). Anthracosis of pulmonary and mesenteric lymph nodes (5/38 animals; 13%) were occasionally present and their frequency increased with age (OR = 1.22, p < 0.0294).

#### Neoplasms

A multiple myeloma affecting the bone marrow, lymph nodes, spleen and pituitary gland was present in a female neutered tiger (animal no. 26). A female cougar had a cutaneous lymphoma dominated by CD3-positive T cells (98%) as determined by immunohistochemistry. A male cheetah (animal no. 1) revealed a myelolipoma within the spleen.

### Cardiovascular system

#### Miscellaneous

Cardiac lipomatosis was detected in 12 of 38 animals (32%) most of which were in good to obese body condition. Mild myocardial fibrosis was identified in 5 animals (13%). The presence of miscellaneous heart findings was not associated with age, sex or specific animal species.

#### Neoplasms

A metastasizing hemangiosarcoma was detected in kidney, liver, bone (right femur) and bone marrow of an 18-year-old female cougar (animal no. 4; Fig 5C) and in the spleen of a 13-year-old female lion (animal no. 14).

### Skeletal system, joints and skin

#### Inflammation

Focal ulcerative dermatitis located at the hindlimb (n = 2) or chronic lympho-histiocytic pododermatitis (n = 2) were present in 4 animals (11%).

#### Degenerative lesions

In 6 of 38 animals (16%) including 4 tigers (animal nos. 25, 29, 31 and 32) and 2 lions (animal nos. 17 and 19), degenerative joint disease affecting one or multiple sites including shoulder, elbow, hip and stifle joint was detected. Lesions were characterized by superficial fibrillation, eburnation, chondrocyte loss, chondrone formation, myelofibrosis, osteosclerosis and proliferation of synovial villi. The risk for the presence of degenerative joint disease was not influenced by age, gender or animal species.

#### Neoplasms

In the 6^th^ lumbar vertebra of a 13-year-old female leopard (animal no. 9), an osteosarcoma was present. In addition, the animal revealed metastases in hepatic and pulmonary lymph nodes, liver, spleen, kidneys, and lung.

## Discussion

The present study aimed to investigate macroscopic and histologic findings in 38 captive wild felids (18 tigers *Panthera tigris*, 8 leopards *Panthera pardus*, 7 lions *Panthera leo*, 3 cheetahs *Acinonyx jubatus* and 2 cougars *Puma concolor*) which died between 2004–2013 in German zoological gardens. The most frequently observed lesions consisted of inflammation and degeneration of the kidney (87%), various neoplasms (50%), enteritis (34%) and pneumonia (32%). In addition, a degenerative encephalopathy in one tiger resembling large felid leukoencephalomyelopathy is described.

### Neoplasms

**Fig 6:**
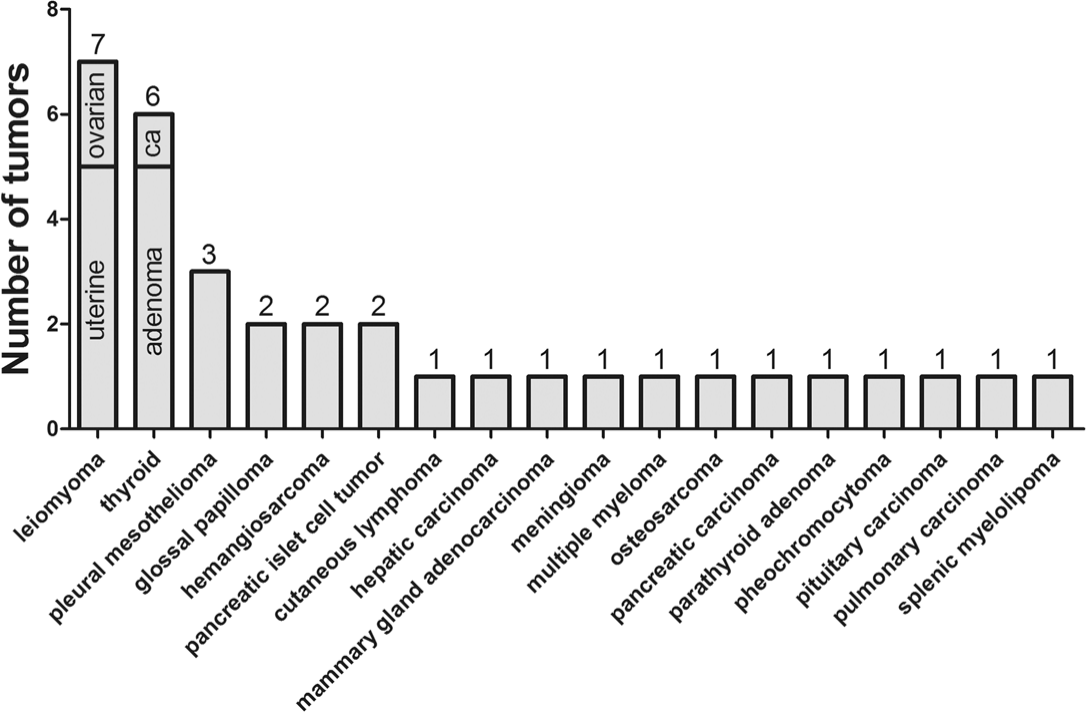
Prevalence of different tumors in 38 captive wild felids.

In the present study, a total of 34 neoplastic lesions in 19 out of 38 animals (50%; Fig 6) with age ranging from 3 to 22 years (median age: 16 years) comprising 13 female and 6 male individuals were observed. In 10 animals including 6 tigers, 2 leopards, 1 lion and 1 cheetah, 2 different tumor types were found. Three different neoplasms were detected in a 17-year-old female leopard (animal no. 13) and 4 distinct tumors were present in an 18-year-old female cougar (animal no. 4). The high prevalence of neoplasms in aged captive felids is similar to observations from a study of the Knoxville zoological garden between 1979–2003 with a tumor rate of 51% [6]. This is in contrast to an earlier study from the Philadelphia zoological garden where the neoplastic rate was much lower and varied from 2.6 to 9.9% between 1901–1934 and 1935–1955, respectively [5]. The differences between the various studies may be related to increased longevity of animals in zoological gardens or environmental factors [5]. However, elevated exposure to carcinogens or infectious agents should be considered as other risk factors [9].

Endocrine glands were most commonly affected by neoplastic lesions in the present study. This was particularly related to the high frequency of thyroid gland tumors such as adenomas and carcinomas in 5 (animals no. 6, 22, 25, 26, 29) and 1 (animal no. 4) animals respectively with a median age of 19 years in affected animals. This finding is in accordance with previous observations from the zoological gardens from Knoxville and Philadelphia that already showed thyroid tumors are common in wild felids [5,6]. In the wild felid population of the Knoxville zoological garden, thyroid tumors are reported in animals with a median age of 18 years [6]. Pathogenetically, an iodine deficiency presumable due to a deficit of the iodine supplementation was associated with a higher incidence of these endocrine tumors [5]. In the present study, it remains undetermined whether similar factors have influenced the development of thyroid neoplasms. In domestic cats, thyroid gland proliferations including hyperplasia and neoplasms are commonly observed with increasing age [39] and affected animals reveal an average age of 12.4 (adenoma) or 15.8 years (carcinoma; [40]). Thyrotoxicosis due to increased production of T3 and T4 by neoplastic cells is a frequent finding in companion animals [39]. Affected domestic cats often have a poor body condition despite polyphagia and might show secondary hypertrophic cardiomyopathy [39,41]. In contrast to domestic cats, wild felids with thyroid hyperplasia or adenoma lacked evidence of thyrotoxicosis consisting of cardiac hypertrophy or emaciation in the present study.

Parathyroid hyperplasia or adenoma was present in 5 animals (animal nos. 4, 5, 7, 25, 27). Concurrent metastatic mineralization of kidneys, lung, bone marrow and/or brain was noticed in 2 of those animals (animal nos. 7 and 25). As both animals further showed renal pathology it remains unresolved whether metastatic mineralization was related to primary or secondary hyperparathyroidism.

A pheochromocytoma was diagnosed in a 19-year-old male tiger (animal no. 27). Although this tumor has not been described in wild felids, it is a rare entity in its domestic counterpart [42]. In companion animals, clinical signs can be attributed to the space-occupying effect of the neoplastic growth [43]. In addition, a pheochromocytoma-associated catecholamine-induced cardiomyopathy is described in dogs, man, nonhuman primates and mice [44]. However, cardiovascular abnormalities were absent in the present study and there was no evidence for chronic congestion indicating that the tumor seemed not to produce significant amounts of systemic active catecholamines.

Genital tumors were second most commonly detected in the present study. Tumors frequently encountered included ovarian and uterine leiomyomas in animals with a median age of 18 years. This finding was similar to previous observations showing genital neoplasms especially leiomyomas of the uterus are frequent in large felids with a median age of 17 years [6]. Although leiomyomas are uncommon in the ovary of domestic cats, they are described as the most common mesenchymal tumor type in the uterus and cats suffering from uterine leiomyomas reveal a median age of 9 years [45,46]. One simple carcinoma of the mammary gland was found in a 13-year-old female leopard (animal no. 9). This is in contrast to other studies reporting a higher prevalence of mammary gland tumors in both wild and domestic felids most likely due to the treatment with contraceptive drugs [6,47,48]. The cause of the low rate of mammary gland tumors in the present study remained undetermined. All female felids (24/38 animals) were treated with contraceptive drugs regularly as mentioned by the referring veterinarians (personal communication).

Lymphoreticular and hematopoietic tumors were the third most commonly occurring type of neoplasms and included multiple myelolipomas in the spleen of one cheetah (animal no. 1). Similarly, myelolipomas in liver (n = 7) or spleen (n = 1) of cheetahs and tigers were described previously [12,49]. A frequency of 6% is reported in captive wild felids and cheetahs revealed the highest prevalence of this tumor type (6/15 cheetahs, 60%) in one study [49]. Pathogenetically, Lombard et al. [49] assumed that these changes represent reactive metaplasias rather than true neoplasms, which may occur as a consequence of anemia. An 18-year-old cougar (animal no. 4) showed a cutaneous T cell lymphoma. Malignant lymphoma is the most common tumor in domestic cats [50] and is occasionally described in lions and rarely reported in cheetahs [8,15,26,51]. Lymphomas in lions most often occur within the spleen and a T cell origin is frequently reported [26]. This is in contrast to domestic cats that show a predominance of B cell neoplasms [26]. Multiple myeloma affecting the bone marrow, lymph nodes, spleen and the pituitary gland was diagnosed in a 19-year-old tiger (animal no. 26). This type of neoplasm was previously only reported in a jaguar [52].

Malignant mesotheliomas were detected in two tigers (animal nos. 21 and 25) and one cheetah (animal no. 1) in the present study. Histologically, they included two biphasic and one epithelioid type. Pleural or pericardial mesotheliomas are rarely described in wild felids including a lion, a clouded leopard and two tigers [10,53–55]. They were graded as malignant tumors with metastasis in one case, and all neoplasms were of the papillary epithelioid type. Pathogenetically, mesotheliomas in man are strongly associated with prolonged exposure to industrial pollutants including asbestos fibers [17,56]. However, no etiology was identified for mesotheliomas in the present study. Similarly, previous observations lacked evidence for intralesional asbestos fibers in affected felids [10,53–55] and therefore other exogenous and endogenous factors have to be considered as potential causes.

Papillomas were detected in the tongue of a 3-year-old lion (animal no. 16) and an 8-year-old tiger (animal no. 24). Roelke et al. [23] reported an association of FIV infection with oral papillomas in African lions. A similar manifestation has been described in humans with immunodeficiency virus infections [57]. However, affected animals lacked evidence for lentiviral infection in the present study. Although papillomas might occur spontaneously, a viral etiology cannot be ruled out.

Hemangiosarcomas were present in the spleen of a 13-year-old female neutered lion (animal no. 14) and in liver, kidney, right femoral bone and bone marrow of an 18-year-old female cougar (animal no. 4). Although these tumors are commonly detected in companion dogs and occasionally observed in domestic cats, no report about their occurrence in wild felids exists [58,59].

A psammomatous meningioma was found in an 18-year-old female cougar (animal no. 4). Meningiomas are common neoplastic lesions in companion cats and are often associated with compression of the adjacent neuroparenchyma [60,61]. In contrast to domestic felids, there is only one report about a meningioma in a 17-year-old Bengal tiger [62].

### Retroviral infection

Retroviral infections such as feline leukemia virus as well as feline immunodeficiency virus are common in domestic cats and have also been described in wild felids [21]. FeLV and FIV infections are associated with neoplastic transformation and can facilitate secondary infections due to immunosuppression, respectively [63,64].

In the present study, FIV specific antibodies were detected in 4 lions (animal nos. 14, 15, 17 and 20), while other animals were negative. Though lymphoid tissues lacked morphologically a loss of lymphocytes a functional impairment of the immune system cannot be ruled out. It is known that FIV infection can promote opportunistic infections and tumor growth [65]. FIV infection was associated with a higher risk for endometritis in the present study. This is in contrast to previous reports where endometritis was not associated with FIV infection in captive wild felids [23,25,66]. Further studies in a larger group of animals are needed to further investigate the possible impact because of the low frequency of both FIV infection and endometritis in the present study. In contrast, the risk for enteritis, pneumonia, metastatic purulent nephritis or neoplasia was not significantly influenced by FIV infection. It has to be considered that this result does not rule out a possible impact of retroviral infection on the development of such lesions because of the limited number of animals and the low prevalence of FIV in the study population. In contrast, FeLV antigen was not detected in any animal in the present study. This is in accordance to previous reports that showed a lower frequency of FeLV infection compared to FIV in non-domestic felids [21].

### Inflammatory and degenerative lesions

Chronic kidney disease represents a common finding in aged domestic and wild felids [16]. In the present study, renal pathology was the most frequent finding with 87% of all investigated captive wild felids. Similarly, previous observations reported a frequency of 74% in non-domestic felids [16]. Chronic renal disease is very frequent in domestic cats with one study reporting a prevalence of up to 30% in aged animals [67,68]. Generally, age is considered to be an important risk factor for the development of chronic renal disease in domestic cats [68,69]. Similarly, the present study demonstrated a higher risk for kidney lesions with increasing age in wild felids. Interstitial nephritis, interstitial fibrosis, intra-tubular concrements, tubular degeneration and necrosis, as well as proteinaceous casts were the most common renal findings in the present study. However, the cause of the renal changes could not be determined. It remains unknown whether the interstitium was primarily or secondarily affected subsequent to tubular damage. In general, infectious and non-infectious etiologies have to be taken into consideration. Besides a hematogenous route, an urogenic-ascending infection as well as a primary glomerular disease have to be considered pathogenetically [16]. Recently, a feline morbillivirus was identified and associated with tubulointerstitial nephritis in a case control study in domestic cats [70]. However, in the present study no morbillivirus antigen was detected in renal lesions.

Increased urea concentration in the anterior eye chamber (> 50 mg/dl) was significantly associated with the presence of concrements in tubular lumina and dilated tubuli but not with other renal lesions, gastritis or enteritis. Impaired renal function may result in uremic gastroenteritis. Azotemia can also be related to pre-renal mechanisms such as dehydration without involvement of renal pathology. However, further studies are needed to substantiate the accuracy of the assumed reference range for wild felids. Despite the absence of an association between renal lesions, gastritis and enteritis, a functional relation of azotemia and renal disease cannot be ruled out. Interstitial nephritis (animal nos. 7 and 25) and azotemia (animal no. 25) were detected in animals with mineralization in kidney, lung and brain suggesting hyperparathyroidism as the underlying pathogenetic mechanism. In addition, elevated levels of parathyroid hormone were observed in 84% of domestic cats with chronic renal failure [71]. Due to the coincidence of parathyroid hyperplasia (animal no. 25) or adenoma (animal no. 7) it remains unknown whether these cases in the present study represent secondary (renal) or primary hyperparathyroidism. Renal inflammation and degeneration were not associated with fibrous osteodystrophy in any case.

Widespread mineralization in various organs including lung, myocardium, kidney, aorta and gastrointestinal tract was also present in 3 young adult tigers (animal nos. 33, 35, 36). Although animal no. 33 showed inflammatory and degenerative renal lesions, these cases were interpreted as metastatic mineralization not initiated by renal or parathyroid gland pathology. In addition, there was no evidence for previous rhabdomyolysis, granulomatous inflammation, osteopathy with accelerated bone turnover or malignant tumors in these animals. Pathogenetically, hypercalcemia due to vitamin D intoxication or idiopathic conditions was suspected in these tigers. In dogs, hypervitaminosis D may occur based on excessive dietary amounts of vitamin D causing clinical signs like lethargy, stiff gait as well as polydipsia in affected animals [72]. Therefore, intoxication with cholecalciferol rodenticides or excessive intake of vitamin D and/or calcium should be considered as possible causes of metastatic mineralization in these tigers.

In wild felids, pneumonia due to various causes including viral, bacterial and fungal agents is occasionally reported [73–75]. Pulmonary inflammation was detected in 12 animals including 6 tigers, 4 lions, 1 leopard and 1 cougar. Intralesional fungal hyphae most likely representing *Aspergillus* sp. were detected in a fibrino-purulent pneumonia in a lion (anima no. 16). Similarly, *Aspergillus terreus* was identified as the cause of the granulomatous pneumonia, rhinitis, encephalitis and thymitis in a neonatal snow leopard [73]. In addition, *Coccidioides immitis* and *Pythium insidiosum* may be considered as further causes of mycotic pneumonia in wild felids, as described in one captive Indochinese tiger and one American jaguar, respectively [76,77]. Interstitial pneumonia represented the most common inflammatory lung lesion in the present study indicating a potential role of viral agents. In wild felids, several viruses are associated with pneumonia including avian influenza A (H5N1) virus (tigers and lions), canine distemper virus (Serengeti lions), FIPV (mountain lion) and FIV (African lion; [25,74,78,79]). Furthermore, feline herpesvirus 1 and feline calicivirus should be considered as potential viral causes of lower respiratory tract disease in wild felids. They represent relevant agents in domestic cats which have also been identified in captive and/or free-ranging cheetahs [1,80]. Fatal cowpox infection induced acute hemorrhagic pneumonia in 2 captive cheetahs [81]. Fibrino-purulent and granulomatous inflammation of the lung detected in the present study suggest a bacterial infection. Several organisms are reported to cause bacterial pneumonia in wild felids comprising extra-intestinal pathogenic *Escherichia coli* (tiger), *Morganella morganii* (jaguar) and *eugonic fermenting bacterium* (African lion; [75,82,83]). Additional special stainings including Gram-, Ziehl-Neelsen staining and PAS reaction did not detect an etiology.No additional tests, like bacterial culture or virological tests to detect infectious agents were carried out in most cases of the present study.

Fourteen animals (34%) suffered from enteritis. Morphologic findings in the small intestine of 6 of these animals shared feature of feline parvovirus infection consisting of crypt dilation with accumulated cellular debris, villous atrophy, fusion and crypt regeneration. Concurrent panmyelophthisis was only present in a 3-year-old Bengal tiger (animal no. 37). However, neither parvovirus- nor FeLV-antigen was detected immunohistologically within the intestinal tract of these animals. FIV antibodies were shown in 2 lions suffering from enteritis (animal nos. 17 and 20). Similarly, chronic enteritis is reported to occur in 19% of FIV-infected domestic cats [24]. Although an impact of FIV infection on the development of enteritis could not be ruled out, the risk for developing enteritis was not increased in infected compared to non-infected felids in the present study. Therefore, a so far unidentified infectious agent must be considered etiologically in the remaining animals.

In contrast to man, the pathogenic role of *Helicobacter* sp. has not been substantiated in most domestic and wild animals and a definitive link to a disease is still lacking except for pigs, ferrets and cheetahs [19,84–88]. *Helicobacter acinonyx*, closely related to *Helicobacter pylori*, is a specific organism described in cheetahs, lions and tigers. This organism is associated with gastritis of varying degree in this species [7,89]. Phylogenetically, the organism is suggested to be derived from *Helicobacter pylori* by crossing the species barrier from early humans to cats within the last 50,000 to 400,000 years [7]. Few genetic modifications determined a better adaptation to the feline immune system [7]. *Helicobacter*-like organisms were demonstrated within the stomach of 3 animals (1 cheetah, 1 cougar and 1 tiger). However, the presence of spirochetes was not associated with gastric inflammation in the present study. This is in contrast to other observations reporting a prevalence of 40% up to 90% for *Helicobacter*-like organisms in captive cheetahs, tigers and lions and infection was associated with increased number of lymphoid follicles and lympho-plasmacytic gastritis [89,90]. *Helicobacter acinonyx* is only reported in captive but not in free roaming wild felids. It is suggested that this may be related to higher baseline cortisol levels in captive animals causing an alteration in their immune response [19].

Degenerative changes of the central nervous system characterized by malacia with internal hydrocephalus ex vacuo, gliosis with prominent gemistocytic astrocytosis and spheroids within the cerebral white matter resembling large felid leukoencephalomyelopathy were present in a tiger (animal no. 29). This entity is described in 73 large captive felids in zoological gardens in the United States and in one animal in the United Kingdom between 1994–2005 [91]. Previous descriptions of large felid leukoencephalomyelopathy reported clinical history of loss of vision with blindness, disorientation and/or difficulty eating. Typically, the disease progressed over days to years [91]. In the present case, similar disturbances regarding vision and eating were not reported. However, the animal showed movement disturbances and recumbency in the final stage. Unfortunately, it was not possible to define the onset and duration of the symptoms. The cause of the disease is unknown although environmental or husbandry-associated sources of neurotoxicity are etiologically suspected. Numerous normal and gemistocytic astrocytes represented characteristic histologic findings in the present and previously reported cases [91]. The present case represents the first occurrence of large felid leukoencephalomyelopathy after 2005 and in Germany.

Non-purulent lymphocytic or lympho-histiocytic meningitis was detected in 3 animals. Pathogenetically this type of lesions can be induced by infectious as well as non-infectious causes. Among the infectious causes viruses like Felid herpesvirus 1, FIPV, FIV as well as bacteria such as *Listeria monocytogenes*, *Klebsiella pneumoniae*, *Streptococcus pneumoniae* and fungi like *Cryptococcus neoformans* have to be considered [92–96]. Though the underlying cause of meningitis remains undetermined in the present study, none of the animals with meningitis were tested positive for FIV.

Dilated myelin sheaths, myelinophagia and spheroids with a mild to moderate degree of severity were detected in 6 animals. Dilated myelin sheaths can result from myelin sheaths edema or represent an early stage of vacuolar degeneration (spongiosis). Myelinophagia can occur during a local or systemic infectious or non-infectious disease with resulting tissue damage. In association with dilated myelin sheaths, spheroids, representing focal swellings of axons, are frequently observed [97]. Few spheroids can commonly be encountered at various sites in brain and spinal cord of older animals and they may not be associated with clinical signs [97].

Neuronal lipofuscinosis, meningeal fibrosis and mineralization (meninges, plexus choroideus, fourth ventricle, hippocampal vessels) were present in the central nervous system (CNS) of few captive wild felids. These lesions were interpreted to be of minor significance and the risk for neuronal lipofuscinosis was significantly increased with age. Similar alterations occur commonly in domestic animals and represent age-related findings in most cases [97].

Male felids revealed a significant higher frequency of meningeal fibrosis compared to female individuals. This is an interesting finding since fibrosis in kidney, heart and lung was not influenced by gender. However, the cause of this sex predisposition is unclear. Further studies are needed to confirm this finding and to investigate possible pathogenetic factors involved in increased meningeal collagen synthesis. Deposition of β-amyloid and neurofibrillary tangles in the brain are described in 13 of 22 captive cheetahs in one study [98]. Similar lesions, representing the hallmark of Alzheimer’s disease in humans, are also described in chimpanzees and wolverines [99–101]. However, in the present study, neither amyloid nor neurofibrillary tangles were detected in the cerebrum of wild felids.

Pyometra and/or endometritis were noticed in two lions, one leopard and one tiger (animal nos. 7, 14, 17 and 24). Pyometra is occasionally reported in wild felids while it is rare in their domestic counterpart [66,102]. Lions seem to be at an increased risk to develop pyometra and this may be related to differences in the induction of ovulation [66]. Similar to its domestic counterparts, *Escherichia coli* appeared to be a more common etiological pathogen than *Pseudomonas aeruginosa* [66,103]. In the present study, the reproductive cycle remains unclear as ovaries were not available due to ovariohysterectomy (n = 1) or not assessed histopathologically (n = 1). Pathogenetically, it remains controversial whether pyometra is promoted by the application of contraceptive drugs or linked to age-related changes associated with endometrial hyperplasia [66,102]. Lympho-histiocytic endometritis with cystic glandular hyperplasia was found in an 8-year-old tiger, which was most likely related to infectious or abiotic factors that may be supported by hormonal disturbances.

In domestic cats, amyloid deposition in pancreatic islets of Langerhans is common and can be detected in 65% of animals with diabetes mellitus [104]. However, islet amyloidosis is also described in approximately 50% of cats that lacked evidence for endocrine disease [105]. In the present study, there was no evidence for amyloidosis in islets of Langerhans within the pancreas in any of the investigated animals.

## Conclusion

In summary, chronic nephropathy characterized by tubular alterations, interstitial nephritis and glomerular lesions was a common finding in captive wild felids. Neoplastic lesions predominantly affecting endocrine, genital, lymphoreticular organs, the pleura as well as the alimentary tract were detected in 50% of the investigated animals. Uterine/ovarian leiomyoma, thyroid gland adenoma/carcinoma, pleural mesothelioma, oral papilloma, hemangiosarcoma and pancreatic islet cell tumor represented the most common tumor types. Several inflammatory and/or degenerative lesions of kidney, lung, intestine, brain as well as the presence of hyperplasia and neoplasia were associated with age of wild felids. Enteritis and pneumonia were the most common inflammatory changes. Large felid leukoencephalomyelopathy is a rare salient lesion which has not been described in Germany before and should be considered as possible differential for degenerative diseases of the CNS. The prevalence of FeLV, FIV and *Helicobacter sp*. in captive wild felids is very low to negligible in German zoological gardens and these organisms seem to play little to no pathogenic role.

## Supporting Information

S1 Table Pathologic lesions in wild captive felids. (XLSX)

## References

[1] Thalwitzer S, Wachter B, Robert N, Wibbelt G, Muller T, Lonzer J, et al Seroprevalences to viral pathogens in free-ranging and captive cheetahs (Acinonyx jubatus) on Namibian Farmland. Clin Vaccine Immunol. 2010;17(2):232–8. 10.1128/CVI.00345-09 19955325 PMC2815525

[2] Henschel P, Coad L, Burton C, Chataigner B, Dunn A, MacDonald D, et al The lion in West Africa is critically endangered. PLoS One. 2014;9(1):e83500 10.1371/journal.pone.0083500 24421889 PMC3885426

[3] IUCN. The IUCN Red List of Threatened Species: International Union for Conservation of Nature and Natural Resources; 2014 [cited 10/19/2014]. Available: http://www.iucnredlist.org/.

[4] Kelly P, Stack D, Harley J. A review of the proposed reintroduction program for the Far Eastern leopard (Panthera pardus orientalis) and the role of conservation organizations, veterinarians, and zoos. Top Companion Anim Med. 2013;28(4):163–6. 10.1053/j.tcam.2013.09.004 24331556

[5] Lombard LS, Witte EJ. Frequency and types of tumors in mammals and birds of the Philadelphia Zoological Garden. Cancer Res. 1959;19(2):127–41. 13629476

[6] Owston MA, Ramsay EC, Rotstein DS. Neoplasia in felids at the Knoxville Zoological Gardens, 1979–2003. J Zoo Wildl Med. 2008;39(4):608–13. 19110704 10.1638/2008-068.1

[7] Eppinger M, Baar C, Linz B, Raddatz G, Lanz C, Keller H, et al Who ate whom? Adaptive Helicobacter genomic changes that accompanied a host jump from early humans to large felines. PLoS Genet. 2006;2(7):e120 16789826 10.1371/journal.pgen.0020120 PMC1523251

[8] Marker L, Munson L, Basson PA, Quackenbush S. Multicentric T-cell lymphoma associated with feline leukemia virus infection in a captive namibian cheetah (Acinonyx jubatus). J Wildl Dis. 2003;39(3):690–5. 14567232 10.7589/0090-3558-39.3.690

[9] McAloose D, Newton AL. Wildlife cancer: a conservation perspective. Nat Rev Cancer. 2009;9(7):517–26. 10.1038/nrc2665 19550426 PMC7096862

[10] Bollo E, Scaglione FE, Tursi M, Schroder C, Degiorgi G, Belluso E, et al Malignant pleural mesothelioma in a female lion (Panthera leo). Res Vet Sci. 2011;91(1):116–8. 10.1016/j.rvsc.2010.08.005 20846704

[11] Doster AR, Armstrong DL, Bargar TW. Seminoma and parathyroid adenoma in a snow leopard (Panthera unica). J Comp Pathol. 1989;100(4):475–80. 2760281 10.1016/0021-9975(89)90016-9

[12] Cardy RH, Bostrom RE. Multiple splenic myelolipomas in a cheetah (Acinonyx jubatus). Vet Pathol. 1978;15(4):556–8. 695230 10.1177/030098587801500414

[13] Wada Y, Kondo H, Bando G, Kosuge M, Ishikawa Y, Kadota K. Intestinal adenocarcinoma with neuroendocrine cells in a clouded leopard (Neofelis nebulosa). J Comp Pathol. 1996;115(3):305–10. 8923240 10.1016/s0021-9975(96)80087-9

[14] Gupta A, Jadav K, Nigam P, Swarup D, Shrivastava AB. Eyelid neoplasm in a white tiger (Panthera tigris)—a case report. Veterinarski arhiv. 2013;83(1):115–24.

[15] Effron M, Griner L, Benirschke K. Nature and rate of neoplasia found in captive wild mammals, birds, and reptiles at necropsy. J Natl Cancer Inst. 1977;59(1):185–98. 577508 10.1093/jnci/59.1.185

[16] Newkirk KM, Newman SJ, White LA, Rohrbach BW, Ramsay EC. Renal lesions of nondomestic felids. Vet Pathol. 2011;48(3):698–705. 10.1177/0300985810382089 20876911

[17] Adams H, van Vuuren M, Bosman AM, Keet D, New J, Kennedy M. The epidemiology of lion lentivirus infection among a population of free-ranging lions (Panthera leo) in the Kruger National Park, South Africa. J S Afr Vet Assoc. 2009;80(3):151–6. 20169747 10.4102/jsava.v80i3.193

[18] Köhler K, Bailey T, Kinne J, Reinacher M. „Feline infektiöse Peritonitis”bei Geparden: Morphologie und Diagnostik. Tierärztliche Praxis Großtiere. 2011;39(2):A2.

[19] Terio KA, Munson L, Moore PF. Characterization of the gastric immune response in cheetahs (Acinonyx jubatus) with Helicobacter-associated gastritis. Vet Pathol. 2012;49(5):824–33. 10.1177/0300985811412620 21730348

[20] Barr MC, Calle PP, Roelke ME, Scott FW. Feline immunodeficiency virus infection in nondomestic felids. J Zoo Wildl Med. 1989;20(3):265–72.

[21] O'Brien SJ, Troyer JL, Brown MA, Johnson WE, Antunes A, Roelke ME, et al Emerging viruses in the Felidae: shifting paradigms. Viruses. 2012;4(2):236–57. 10.3390/v4020236 22470834 PMC3315214

[22] Roelke ME, Pecon-Slattery J, Taylor S, Citino S, Brown E, Packer C, et al T-lymphocyte profiles in FIV-infected wild lions and pumas reveal CD4 depletion. J Wildl Dis. 2006;42(2):234–48. 16870846 10.7589/0090-3558-42.2.234

[23] Roelke ME, Brown MA, Troyer JL, Winterbach H, Winterbach C, Hemson G, et al Pathological manifestations of feline immunodeficiency virus (FIV) infection in wild African lions. Virology. 2009;390(1):1–12. 10.1016/j.virol.2009.04.011 19464039 PMC2771374

[24] Yamamoto JK, Hansen H, Ho EW, Morishita TY, Okuda T, Sawa TR, et al Epidemiologic and clinical aspects of feline immunodeficiency virus infection in cats from the continental United States and Canada and possible mode of transmission. J Am Vet Med Assoc. 1989;194(2):213–20. 2537269

[25] Poli A, Abramo F, Cavicchio P, Bandecchi P, Ghelardi E, Pistello M. Lentivirus infection in an African lion: a clinical, pathologic and virologic study. J Wildl Dis. 1995;31(1):70–4. 7563428 10.7589/0090-3558-31.1.70

[26] Harrison TM, McKnight CA, Sikarskie JG, Kitchell BE, Garner MM, Raymond JT, et al Malignant lymphoma in african lions (panthera leo). Vet Pathol. 2010;47(5):952–7. 10.1177/0300985810375054 20610770

[27] Citino SB. Transient FeLV Viremia in a Clouded Leopard. J Zoo Wildl Med. 1986;17(1):5–7.

[28] Brown MA, Cunningham MW, Roca AL, Troyer JL, Johnson WE, O'Brien SJ. Genetic characterization of feline leukemia virus from Florida panthers. Emerg Infect Dis. 2008;14(2):252–9. 10.3201/eid1402.070981 18258118 PMC2600209

[29] Cunningham MW, Brown MA, Shindle DB, Terrell SP, Hayes KA, Ferree BC, et al Epizootiology and management of feline leukemia virus in the Florida puma. J Wildl Dis. 2008;44(3):537–52. 18689639 10.7589/0090-3558-44.3.537 PMC3167064

[30] Mulisch M, Welsch U, editors. Romeis—Mikroskopische Technik 18th ed. Heidelberg, Germany: Spektrum Akademischer Verlag; 2010.

[31] Seehusen F, Orlando EA, Wewetzer K, Baumgärtner W. Vimentin-positive astrocytes in canine distemper: a target for canine distemper virus especially in chronic demyelinating lesions? Acta Neuropathol. 2007;114(6):597–608. 17965866 10.1007/s00401-007-0307-5

[32] Hanna PE, Bellamy JE, Donald A. Postmortem eyefluid analysis in dogs, cats and cattle as an estimate of antemortem serum chemistry profiles. Can J Vet Res. 1990;54(4):487–94. 2249181 PMC1255698

[33] Lane VM, Lincoln SD. Changes in urea nitrogen and creatinine concentrations in the vitreous humor of cattle after death. Am J Vet Res. 1985;46(7):1550–2. 4026038

[34] McLaughlin BG, McLaughlin PS. Equine vitreous humor chemical concentrations: correlation with serum concentrations, and postmortem changes with time and temperature. Can J Vet Res. 1988;52(4):476–80. 3196976 PMC1255494

[35] Schoning P, Strafuss AC. Postmortem biochemical changes in canine vitreous humor. Journal of forensic sciences. 1980;25(1):53–9. 7391783

[36] R Core Team. R: A Language and Environment for Statistical Computing Vienna, Austria: R Foundation for Statistical Computing; 2014 [cited 10/19/2014]. Available: http://www.R-project.org/.

[37] Landis JR, Koch GG. The measurement of observer agreement for categorical data. Biometrics. 1977;33(1):159–74. 843571

[38] Gerhauser I, Philipp U, Distl O, Beineke A. Multiple cyst formation in the liver and kidneys of a lion (Panthera leo): a case of polycystic kidney disease? Eur J Wildl Res. 2009;55(4):433–7.

[39] Peterson M. Hyperthyroidism in cats: what's causing this epidemic of thyroid disease and can we prevent it? J Feline Med Surg. 2012;14(11):804–18. 10.1177/1098612X12464462 23087006 PMC11112171

[40] Leav I, Schiller AL, Rijnberk A, Legg MA, der Kinderen PJ. Adenomas and carcinomas of the canine and feline thyroid. Am J Pathol. 1976;83(1):61–122. 776005 PMC2032435

[41] Liu SK, Peterson ME, Fox PR. Hypertropic cardiomyopathy and hyperthyroidism in the cat. J Am Vet Med Assoc. 1984;185(1):52–7. 6540256

[42] Calsyn JD, Green RA, Davis GJ, Reilly CM. Adrenal pheochromocytoma with contralateral adrenocortical adenoma in a cat. Journal of the American Animal Hospital Association. 2010;46(1):36–42. 20045835 10.5326/0460036

[43] Maher ER Jr., McNiel EA. Pheochromocytoma in dogs and cats. Vet Clin North Am Small Anim Pract. 1997;27(2):359–80. 9076913 10.1016/s0195-5616(97)50037-4

[44] Edmondson EF, Bright JM, Halsey CH, Ehrhart EJ. Pathologic and cardiovascular characterization of pheochromocytoma-associated cardiomyopathy in dogs. Vet Pathol. 2015;52(2):338–43. 10.1177/0300985814533805 24810909

[45] Gelberg HB, McEntee K. Feline ovarian neoplasms. Vet Pathol. 1985;22(6):572–6. 4082383 10.1177/030098588502200610

[46] Miller MA, Ramos-Vara JA, Dickerson MF, Johnson GC, Pace LW, Kreeger JM, et al Uterine neoplasia in 13 cats. J Vet Diagn Invest. 2003;15(6):515–22. 14667013 10.1177/104063870301500602

[47] Harrenstien LA, Munson L, Seal US. Mammary Cancer in Captive Wild Felids and Risk Factors for Its Development: A Retrospective Study of the Clinical Behavior of 31 Cases. J Zoo Wildl Med. 1996;27(4):468–76.

[48] Munson L, Moresco A. Comparative pathology of mammary gland cancers in domestic and wild animals. Breast Dis. 2007;28:7–21. 18057539 10.3233/bd-2007-28102

[49] Lombard LS, Fortna HM, Garner FM, Brynjolfsson G. Myelolipomas of the liver in captive wild Felidae. Pathol Vet. 1968;5(2):127–34. 5691491 10.1177/030098586800500204

[50] Vail DM, Moore AS, Ogilvie GK, Volk LM. Feline lymphoma (145 cases): proliferation indices, cluster of differentiation 3 immunoreactivity, and their association with prognosis in 90 cats. J Vet Intern Med. 1998;12(5):349–54. 9773411 10.1111/j.1939-1676.1998.tb02134.x

[51] Harrison TM, Sikarskie J, Kitchell B, Rosenstein DS, Flaherty H, Fitzgerald SD, et al Treatment of malignant lymphoma in an African lion (Panthera leo). J Zoo Wildl Med. 2007;38(2):333–6. 17679520 10.1638/1042-7260(2007)038[0333:TOMLIA]2.0.CO;2

[52] Port CD, Maschgan ER, Pond J, Scarpelli DG. Multiple Neoplasia in a jaguar (Panthera onca). J Comp Pathol. 1981;91(1):115–22. 7343568 10.1016/0021-9975(81)90051-7

[53] Cunningham AA, Dhillon AP. Pleural malignant mesothelioma in a captive clouded leopard (Neofelis nebulosa nebulosa). Vet Rec. 1998;143(1):22–4. 9698630 10.1136/vr.143.1.22

[54] Shin NS, Kwon SW, Kim DY, Kweon OK, Seo IB, Kim JH. Metastatic malignant mesothelioma in a tiger (Panthera tigris). J Zoo Wildl Med. 1998;29(1):81–3. 9638633

[55] Wiedner EB, Isaza R, Lindsay WA, Case AL, Decker J, Roberts J. Pericardial mesothelioma in a Bengal tiger (Panthera tigris). J Zoo Wildl Med. 2008;39(1):121–3. 18432108 10.1638/2007-0080.1

[56] Adams H, van Vuuren M, Kania S, Bosman AM, Keet D, New J, et al Sensitivity and specificity of a nested polymerase chain reaction for detection of lentivirus infection in lions (Panthera leo). J Zoo Wildl Med. 2010;41(4):608–15. 21370640 10.1638/2009-0137.1

[57] van der Burg SH, Palefsky JM. Human Immunodeficiency Virus and Human Papilloma Virus—why HPV-induced lesions do not spontaneously resolve and why therapeutic vaccination can be successful. Journal of translational medicine. 2009;7:108 10.1186/1479-5876-7-108 20021658 PMC2802355

[58] MacEwen EG. Spontaneous tumors in dogs and cats: models for the study of cancer biology and treatment. Cancer Metastasis Rev. 1990;9(2):125–36. 2253312 10.1007/BF00046339

[59] Schultheiss PC. A retrospective study of visceral and nonvisceral hemangiosarcoma and hemangiomas in domestic animals. J Vet Diagn Invest. 2004;16(6):522–6. 15586567 10.1177/104063870401600606

[60] Motta L, Mandara MT, Skerritt GC. Canine and feline intracranial meningiomas: an updated review. Vet J. 2012;192(2):153–65. 10.1016/j.tvjl.2011.10.008 22104505

[61] Troxel MT, Vite CH, Van Winkle TJ, Newton AL, Tiches D, Dayrell-Hart B, et al Feline intracranial neoplasia: retrospective review of 160 cases (1985–2001). J Vet Intern Med. 2003;17(6):850–9. 14658723 10.1111/j.1939-1676.2003.tb02525.x

[62] Akin EY, Baumgartner WA, Lee JK, Beasley MJ. Meningioma in a Bengal tiger (Panthera tigris tigris). J Zoo Wildl Med. 2013;44(3):761–4. 24063109 10.1638/2012-0215R.1

[63] Beatty J. Viral causes of feline lymphoma: Retroviruses and beyond. Vet J. 2014;201(2):174–80. 10.1016/j.tvjl.2014.05.026 24928422

[64] Magden E, Quackenbush SL, VandeWoude S. FIV associated neoplasms—a mini-review. Vet Immunol Immunopathol. 2011;143(3–4):227–34. 10.1016/j.vetimm.2011.06.035 21722968

[65] Hartmann K. Clinical aspects of feline immunodeficiency and feline leukemia virus infection. Vet Immunol Immunopathol. 2011;143(3–4):190–201. 10.1016/j.vetimm.2011.06.035 21807418 PMC7132395

[66] McCain S, Ramsay E, Allender MC, Souza C, Schumacher J. Pyometra in captive large felids: a review of eleven cases. J Zoo Wildl Med. 2009;40(1):147–51. 19368254 10.1638/2008-0008.1

[67] Lulich JP, Osborne CA, Obrien TD, Polzin DJ. Feline renal failure: Questions, answers, questions. Comp Cont Educ Pract. 1992;14(2):127-&.

[68] Reynolds BS, Lefebvre HP. Feline CKD: Pathophysiology and risk factors—what do we know? J Feline Med Surg. 2013;15 Suppl 1:3–14. 10.1177/1098612X13495234 23999182 PMC10816689

[69] Elliott J, Barber PJ. Feline chronic renal failure: clinical findings in 80 cases diagnosed between 1992 and 1995. J Small Anim Pract. 1998;39(2):78–85. 9513888 10.1111/j.1748-5827.1998.tb03598.x

[70] Woo PC, Lau SK, Wong BH, Fan RY, Wong AY, Zhang AJ, et al Feline morbillivirus, a previously undescribed paramyxovirus associated with tubulointerstitial nephritis in domestic cats. Proc Natl Acad Sci U S A. 2012;109(14):5435–40. 10.1073/pnas.1119972109 22431644 PMC3325679

[71] Barber PJ, Elliott J. Feline chronic renal failure: calcium homeostasis in 80 cases diagnosed between 1992 and 1995. J Small Anim Pract. 1998;39(3):108–16. 9551377 10.1111/j.1748-5827.1998.tb03613.x

[72] Mellanby RJ, Mee AP, Berry JL, Herrtage ME. Hypercalcaemia in two dogs caused by excessive dietary supplementation of vitamin D. J Small Anim Pract. 2005;46(7):334–8. 16035450 10.1111/j.1748-5827.2005.tb00329.x

[73] Peden WM, Richard JL, Trampel DW, Brannian RE. Mycotic pneumonia and meningoencephalitis due to Aspergillus terreus in a neonatal snow leopard (Panthera uncia). J Wildl Dis. 1985;21(3):301–5. 4032629 10.7589/0090-3558-21.3.301

[74] Keawcharoen J, Oraveerakul K, Kuiken T, Fouchier RA, Amonsin A, Payungporn S, et al Avian influenza H5N1 in tigers and leopards. Emerg Infect Dis. 2004;10(12):2189–91. 15663858 10.3201/eid1012.040759 PMC3323383

[75] Carvallo FR, Debroy C, Baeza E, Hinckley L, Gilbert K, Choi SJ, et al Necrotizing pneumonia and pleuritis associated with extraintestinal pathogenic Escherichia coli in a tiger (Panthera tigris) cub. J Vet Diagn Invest. 2010;22(1):136–40. 20093704 10.1177/104063871002200130

[76] Camus AC, Grooters AM, Aquilar RE. Granulomatous pneumonia caused by Pythium insidiosum in a central American jaguar, Panthera onca. J Vet Diagn Invest. 2004;16(6):567–71. 15586573 10.1177/104063870401600612

[77] Helmick KE, Koplos P, Raymond J. Disseminated coccidioidomycosis in a captive Indochinese tiger (Panthera tigris corbetti) with chronic renal disease. J Zoo Wildl Med. 2006;37(4):542–4. 17315442 10.1638/04-042.1

[78] Stephenson N, Swift P, Moeller RB, Worth SJ, Foley J. Feline infectious peritonitis in a mountain lion (Puma concolor), California, USA. J Wildl Dis. 2013;49(2):408–12. 10.7589/2012-08-210 23568918

[79] Roelke-Parker ME, Munson L, Packer C, Kock R, Cleaveland S, Carpenter M, et al A canine distemper virus epidemic in Serengeti lions (Panthera leo). Nature. 1996;379(6564):441–5. 8559247 10.1038/379441a0 PMC7095363

[80] Binns SH, Dawson S, Speakman AJ, Cuevas LE, Hart CA, Gaskell CJ, et al A study of feline upper respiratory tract disease with reference to prevalence and risk factors for infection with feline calicivirus and feline herpesvirus. J Feline Med Surg. 2000;2(3):123–33. 11716607 10.1053/jfms.2000.0084 PMC10829116

[81] Baxby D, Ashton DG, Jones DM, Thomsett LR. An outbreak of cowpox in captive cheetahs: virological and epidemiological studies. J Hyg (Lond). 1982;89(3):365–72. 6891393 10.1017/s0022172400070935 PMC2134230

[82] Choi JH, Yoo HS, Park JY, Kim YK, Kim E, Kim DY. Morganelliasis pneumonia in a captive jaguar. J Wildl Dis. 2002;38(1):199–201. 11838216 10.7589/0090-3558-38.1.199

[83] Fenwick BW, Jang SS, Gillespie DS. Pneumonia caused by a eugonic fermenting bacterium in an African lion. J Am Vet Med Assoc. 1983;183(11):1315–7. 6643258

[84] Simpson KW. Diseases of the Gastrointestinal Tract: Stomach In: Washabau RJ, Day MJ, editors. Canine and Feline Gastroenterology. Saint Louis: W.B. Saunders; 2013 p. 606–50.

[85] Schrenzel MD, Witte CL, Bahl J, Tucker TA, Fabian N, Greger H, et al Genetic characterization and epidemiology of Helicobacters in non-domestic animals. Helicobacter. 2010;15(2):126–42. 10.1111/j.1523-5378.2009.00744.x 20402815

[86] Bracarense AP, Yamasaki L, Silva EO, Oliveira RL, Alfieri AA. Helicobacter spp. infection induces changes in epithelial proliferation and E-cadherin expression in the gastric mucosa of pigs. J Comp Pathol. 2013;149(4):402–9. 10.1016/j.jcpa.2013.06.002 24011902

[87] Erdman SE, Correa P, Coleman LA, Schrenzel MD, Li X, Fox JG. Helicobacter mustelae-associated gastric MALT lymphoma in ferrets. Am J Pathol. 1997;151(1):273–80. 9212752 PMC1857920

[88] Fox JG, Dangler CA, Sager W, Borkowski R, Gliatto JM. Helicobacter mustelae-associated gastric adenocarcinoma in ferrets (Mustela putorius furo). Vet Pathol. 1997;34(3):225–9. 9163879 10.1177/030098589703400308

[89] Jakob W, Stolte M, Valentin A, Schroder HD. Demonstration of Helicobacter pylori-like organisms in the gastric mucosa of captive exotic carnivores. J Comp Pathol. 1997;116(1):21–33. 9076597 10.1016/s0021-9975(97)80040-0

[90] Munson L, Nesbit JW, Meltzer DG, Colly LP, Bolton L, Kriek NP. Diseases of captive cheetahs (Acinonyx jubatus jubatus) in South Africa: a 20-year retrospective survey. J Zoo Wildl Med. 1999;30(3):342–7. 10572855

[91] Brower AI, Munson L, Radcliffe RW, Citino SB, Lackey LB, Van Winkle TJ, et al Leukoencephalomyelopathy of mature captive cheetahs and other large felids: a novel neurodegenerative disease that came and went? Vet Pathol. 2014;51(5):1013–21. 10.1177/0300985813506917 24129896

[92] Abramo F, Bo S, Canese MG, Poli A. Regional distribution of lesions in the central nervous system of cats infected with feline immunodeficiency virus. AIDS Res Hum Retroviruses. 1995;11(10):1247–53. 8573382 10.1089/aid.1995.11.1247

[93] Hochwald GM, Nakamura S, Chase R, Gorelick J. Cerebrospinal fluid glucose and leukocyte responses in experimental meningitis. J Neurol Sci. 1984;63(3):381–91. 6374041 10.1016/0022-510x(84)90161-8

[94] Hora AS, Tonietti PO, Guerra JM, Leme MC, Pena HFJ, Maiorka PC, et al Felid Herpesvirus 1 as a Causative Agent of Severe Nonsuppurative Meningoencephalitis in a Domestic Cat. J Clin Microbiol. 2013;51(2):676–9. 10.1128/JCM.02462-12 23152556 PMC3553892

[95] Pal M. Feline Meningitis Due to Cryptococcus-Neoformans Var Neoformans and Review of Feline Cryptococcosis. Mycoses. 1991;34(7–8):313–6. 1803233 10.1111/j.1439-0507.1991.tb00666.x

[96] Poncelet L, Coppens A, Peeters D, Bianchi E, Grant CK, Kadhim H. Detection of antigenic heterogeneity in feline coronavirus nucleocapsid in feline pyogranulomatous meningoencephalitis. Vet Pathol. 2008;45(2):140–53. 10.1354/vp.45-2-140 18424826

[97] Wohlsein P, Deschl U, Baumgärtner W. Nonlesions, unusual cell types, and postmortem artifacts in the central nervous system of domestic animals. Vet Pathol. 2013;50(1):122–43. 10.1177/0300985812450719 22692622

[98] Serizawa S, Chambers JK, Une Y. Beta amyloid deposition and neurofibrillary tangles spontaneously occur in the brains of captive cheetahs (Acinonyx jubatus). Vet Pathol. 2012;49(2):304–12. 10.1177/0300985811410719 21712514

[99] Braak H, Braak E. Staging of Alzheimer's disease-related neurofibrillary changes. Neurobiol Aging. 1995;16(3):271–8; discussion 8–84. 7566337 10.1016/0197-4580(95)00021-6

[100] Roertgen KE, Parisi JE, Clark HB, Barnes DL, O'Brien TD, Johnson KH. A beta-associated cerebral angiopathy and senile plaques with neurofibrillary tangles and cerebral hemorrhage in an aged wolverine (Gulo gulo). Neurobiol Aging. 1996;17(2):243–7. 8744405 10.1016/0197-4580(95)02069-1

[101] Rosen RF, Farberg AS, Gearing M, Dooyema J, Long PM, Anderson DC, et al Tauopathy with paired helical filaments in an aged chimpanzee. J Comp Neurol. 2008;509(3):259–70. 10.1002/cne.21744 18481275 PMC2573460

[102] Munson L, Gardner A, Mason RJ, Chassy LM, Seal US. Endometrial hyperplasia and mineralization in zoo felids treated with melengestrol acetate contraceptives. Vet Pathol. 2002;39(4):419–27. 12126144 10.1354/vp.39-4-419

[103] Agudelo CF. Cystic endometrial hyperplasia-pyometra complex in cats. A review. Vet Q. 2005;27(4):173–82. 16402514

[104] Yano BL, Hayden DW, Johnson KH. Feline insular amyloid: association with diabetes mellitus. Vet Pathol. 1981;18(5):621–7. 7025435 10.1177/030098588101800507

[105] Yano BL, Hayden DW, Johnson KH. Feline insular amyloid: incidence in adult cats with no clinicopathologic evidence of overt diabetes mellitus. Vet Pathol. 1981;18(3):310–5. 7020225 10.1177/030098588101800303

